# Comparative Secretome Analysis of *Magnaporthe oryzae* Identified Proteins Involved in Virulence and Cell Wall Integrity

**DOI:** 10.1016/j.gpb.2021.02.007

**Published:** 2021-07-18

**Authors:** Ning Liu, Linlu Qi, Manna Huang, Deng Chen, Changfa Yin, Yiying Zhang, Xingbin Wang, Guixin Yuan, Rui-Jin Wang, Jun Yang, You-Liang Peng, Xunli Lu

**Affiliations:** 1 State Key Laboratory of Agrobiotechnology and MOA Key Laboratory of Pest Monitoring and Green Management, China Agricultural University, Beijing 100193, China; 2 MOA Key Laboratory of Pest Monitoring and Green Management, College of Plant Protection, China Agricultural University, Beijing 100193, China; 3 Graduate School of China Agricultural University, Beijing 100193, China

**Keywords:** *Magnaporthe oryzae*, Secretome, *N*-glycosylation, Invertase 1, Acid mammalian chinitase

## Abstract

Plant fungal pathogens secrete numerous proteins into the apoplast at the plant–fungus contact sites to facilitate colonization. However, only a few secretory proteins were functionally characterized in *Magnaporthe oryzae*, the fungal pathogen causing rice blast disease worldwide. Asparagine-linked glycosylation 3 (Alg3) is an α-1,3-mannosyltransferase functioning in the *N*-glycan synthesis of *N*-glycosylated secretory proteins. Fungal pathogenicity and cell wall integrity are impaired in Δ*alg3* mutants, but the secreted proteins affected in Δ*alg3* mutants are largely unknown. In this study, we compared the **secretomes** of the wild-type strain and the Δ*alg3* mutant and identified 51 proteins that require Alg3 for proper secretion. These proteins were predicted to be involved in metabolic processes, interspecies interactions, cell wall organization, and response to chemicals. Nine proteins were selected for further validation. We found that these proteins were localized at the apoplastic region surrounding the fungal infection hyphae. Moreover, the ***N*-glycosylation** of these proteins was significantly changed in the Δ*alg3* mutant, leading to the decreased protein secretion and abnormal protein localization. Furthermore, we tested the biological functions of two genes, *INV1* (encoding **invertase 1**, a secreted invertase) and *AMCase* (encoding **acid mammalian chinitase**, a secreted chitinase). The fungal virulence was significantly reduced, and the cell wall integrity was altered in the Δ*inv1* and Δ*amcase* mutant strains. Moreover, the *N*-glycosylation was essential for the function and secretion of AMCase. Taken together, our study provides new insight into the role of *N*-glycosylated secretory proteins in fungal virulence and cell wall integrity.

## Introduction

The fungal pathogen *Magnaporthe oryzae* causes rice blast disease, one of the most devastating diseases of cultivated rice (*Oryza sativa*) worldwide [1]. Due to its agronomic and scientific importance, *M. oryzae* has become a model fungus to study plant–pathogen interactions. As a hemibiotrophic fungus, *M. oryzae* undergoes an initial biotrophic infection stage and then switches to a necrotrophic stage [2], [3]. Once in contact with the plant leaf, the conidium germinates, and the germ tip forms a dome-shaped appressorium on the leaf surface. The appressorium matures and develops a penetration peg to rupture the plant cuticle and invade the epidermal cells. After penetration, the fungus develops biotrophic invasive hyphae in the initial leaf cell and branches into neighboring cells. Eventually, the fungus generates a visible necrotic lesion with numerous, newly formed conidia ready for the next infection cycle [4]. *M. oryzae* mutants impaired in appressorium and/or infection hyphae formation also show defects in pathogenicity, indicating that fungal appressorium formation and biotrophic growth are essential for successful infection [5], [6], [7], [8], [9], [10]. Therefore, exploring the molecular basis for these processes enables us to illustrate the mechanisms underlying plant–fungus interactions and develop new anti-fungal strategies.

During the initial biotrophic infection stage, *M. oryzae* secretes numerous proteins into the plant–fungus contact sites to facilitate colonization. One class of those secreted proteins are enzymes, including cutinase 2, endoglucanase, endo-β-1,4 xylanase, and cellulases that break down the plant cell wall; the other class of secreted proteins is small effector proteins that suppress the host immune system or manipulate host metabolism [11], [12], [13], [14], [15]. *M. oryzae* effector proteins include cytoplasmic effectors and apoplastic effectors, which are secreted through distinct pathways [16]. The cytoplasmic effectors, such as pathogenicity toward weeping lovegrass (PWL2) and biotrophy-associated secreted protein 1 (BAS1), are secreted through exocyst components to the biotrophic interfacial complex, a specific plant membrane-rich structure associated with invasive hyphae. Many cytoplasmic effector proteins have been studied, especially numerous avirulent effector proteins, including AvrPia, Avr1-CO39, AvrPita, AvrPik, and AvrPiz-t [17], [18], [19], [20], [21], [22], [23]. The apoplastic effectors, such as secreted lysM protein 1 (Slp1) and biotrophy-associated secreted protein 4 (BAS4), are secreted from invasive hyphae into the extracellular compartment surrounding invasive hyphae via the conventional secretory pathway from the endoplasmic reticulum (ER) to the Golgi apparatus [8], [12], [16]. So far, only a few *M. oryzae* apoplastic effector proteins have been characterized. The virulence effector Slp1 functions in host immune suppression by binding chitin oligosaccharides to avoid the chitin-induced plant immune response [12]. Since proteins secreted at early biotrophic fungal growth stages contribute to fungal pathogenicity, characterization of additional secretory proteins during invasive hyphae growth may help us to better understand the mechanisms of the rice–*M. oryzae* interaction.

*N*-glycosylation of proteins is a post-translational modification commonly found in eukaryotic organisms. Many *N*-glycosylated proteins are plasma membrane-associated proteins or secreted proteins. The proper folding of these proteins relies on the correct *N*-glycosylation process, which is related to a proper secretion route to their functional sites. *N*-glycosylation starts with the synthesis of the core oligosaccharide NAcGlc_2_Man_9_Glc_3_ — short for two *N*-acetylglucosamines (NAcGlc), nine mannoses (Man), and three glucose (Glc) molecules. After that, the core oligosaccharide is added to an asparagine residue (*N*) in the consensus sequence Asn-x-Ser/Thr (x is any amino acid apart from proline). The *N*-glycosylation modification takes place in the ER and Golgi apparatus, following the conventional secretion route for those target proteins. Defects in the early step of *N*-glycosylation result in improper folding of target proteins, leading to protein breakdown through ER-associated degradation. Correct *N*-glycosylation is necessary for the proper functions of secreted proteins in eukaryotes. For instance, impaired plant *N*-glycosylation or quality control for glycoprotein folding in the ER results in reduced plant resistance to bacterial pathogens [24], [25]. In pathogenic fungi, defects of *N*-glycosylation modification result in a reduction of fungal pathogenicity [26], [27]. In *M. oryzae*, *ALG3* encodes asparagine-linked glycosylation 3, an α-1,3-mannosyltransferase that functions in core oligosaccharide synthesis. Deletion of *ALG3* results in a significant reduction of fungal virulence and defects in fungal cell wall integrity. The apoplastic effector Slp1 is the target protein requiring Alg3 for its proper *N*-glycosylation [28]. Δ*alg3* exhibits a much stronger phenotype than that of the loss-of-function Δ*slp1* mutant; this indicates that apart from Slp1, other target proteins should also be affected in the Δ*alg3* mutant. Therefore, we took a proteomics approach to identify other secretory proteins affected in the Δ*alg3* mutant, which should play essential roles in fungal virulence and cell wall integrity.

In this study, we performed a comparative secretome analysis to identify secreted *N*-glycosylated proteins whose secretion requires functional Alg3. Comparing the *in vitro* secretomes of *M*. *oryzae* wild-type strain P131 and the knockout mutant Δ*alg3*, we found 51 proteins that were not secreted or secreted in reduced amounts in the Δ*alg3* mutant. We confirmed that for 9 out of those 51 proteins, their *N*-glycosylation levels and their localization in the infection hyphae were affected in the Δ*alg3* mutant. We confirmed that two genes, *INV1* (encoding invertase 1) and *AMCase* (encoding acid mammalian chinitase), function in fungal pathogenicity and cell wall integrity. Our study provides new insight into the role of secreted *N*-glycosylated proteins in fungal virulence and cell wall integrity.

## Results

### Identification of *M. oryzae* secreted proteins requiring Alg3 for proper secretion

**Figure 1:**
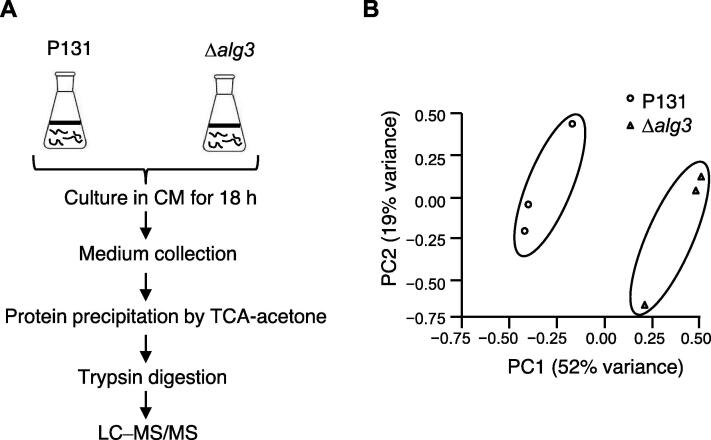
**Quantitative secretome analysis of *Magnaporthe oryzae* secreted proteins from wild-type P131 and Δ*alg3* mutant strains** **A.** Experimental strategy for identifying secreted proteins of *M. oryzae* from the liquid medium. **B.** PCA indicated that the identified proteins from wild-type P131 strains and *Δalg3* strains were grouped separately. CM, complete medium; TCA, trichloroacetic acid; LC–MS/MS, liquid chromatography–tandem mass spectrometry; PCA, principal component analysis; PC, principal component.

In order to identify *N*-glycosylated proteins that require Alg3 for their secretion, we collected *M. oryzae* secreted proteins from the wild-type strain P131 and the knockout mutant Δ*alg3* grown in a liquid growth medium (Figure 1A). *M. oryzae* may experience nutrient-deficient conditions when infecting susceptible plants; therefore, to better mimic the nutrient-deficient conditions during *M. oryzae* infection *in vitro*, we first compared the secreted proteins isolated from hyphae grown on nutrient-sufficient complete medium (CM) and nutrient-deficient minimal medium (MM). However, immunoblotting analysis using GFP fusion proteins found that Slp1, a known effector protein whose secretion is regulated by Alg3 [28], was only detected from *M. oryzae* grown in liquid CM but not from cultures grown in liquid MM (Figure S1A). Therefore, secreted proteins were collected from *M. oryzae* grown in liquid CM. Three replicates of extracted P131 and Δ*alg3* protein samples were separated by sodium dodecyl sulfate–polyacrylamide gel electrophoresis (SDS-PAGE), and protein bands were clearly detected using silver staining (Figure S1B), indicating that those samples were suitable for subsequent analysis. The same amounts of proteins were then used for liquid chromatography–tandem mass spectrometry (LC–MS/MS) analysis.

Using a label-free quantitative (LFQ) proteomic analysis, we identified 411 proteins from the wild-type P131 strain and 539 proteins from the Δ*alg3* mutant strain (Table S1). The replication ratio was > 74% for both samples (Table 1, Table S1). We used the principal component analysis (PCA) to analyze the correlation and distribution of those secreted proteins from P131 and Δ*alg3* samples based on the protein intensity. In the PCA plot, P131 samples were clearly separated from Δ*alg3* samples (Figure 1B), confirming that the isolated secreted proteins are different between P131 and Δ*alg3* strains.

**Table 1:** **An overview of the identified secreted proteins of *Magnaporthe oryzae* from three biological replicates**

**Parameter**	**P131**	**Δ*alg3***
No. of proteins identified in R1	360	471
No. of proteins identified in R2	276	503
No. of proteins identified in R3	310	455
No. of proteins commonly identified in R1, R2, and R3	233	371
Replication rate (%)	74.77	78.02

*Note*: Replication rate was calculated as the average percentage of proteins commonly identified in all three replicates among the proteins identified in individual replicates. R, replicate.

Based on the protein LFQ intensities, we calculated the ratio of proteins in P131 to Δ*alg3*, and selected the proteins with the intensity ratio > 2.0 or < 0.5 (*P* < 0.05; Student’s *t*-test). The identified proteins could be classified into three groups: 1) Group 1, including 51 proteins with a significantly higher abundance in the secretome of P131 than in Δ*alg3*; 2) Group 2, including 295 proteins with no significant difference in abundance between two secretomes; and 3) Group 3, including 210 proteins with a significant higher abundance in the secretome of Δ*alg3* than in P131 (Table 2, Table S1). In addition, we corrected the *P* values with the Benjamini–Hochberg correction, and 8 proteins passed the FDR correction (Table S1). Alg3 is an *α*-1,3-mannosyltransferase, which functions in the biosynthesis of the core oligosaccharide of *N*-glycan, and loss of Alg3 alters protein *N*-glycosylation modifications and reduces client protein steady-state levels, as shown previously for the effector protein Slp1. Therefore, proteins with a significantly lower abundance in Δ*alg3* (Group 1 proteins) are putative Alg3-regulated secretory proteins (Table 3).

**Table 2:** **An overview of the secreted proteins in different groups**

**Parameter**	**Group 1**	**Group 2**	**Group 3**
No. of total proteins identified	51	295	210
No. of proteins predicted by SignalP	43	128	33
No. of proteins predicted by SecretomeP	7	69	43
No. of proteins predicted by TMHMM	11	22	14

*Note*: Protein abundance was calculated based on LFQ intensity. Group 1 included the proteins that were not detected in Δ*alg3* or the proteins showing a significantly higher abundance in P131 with intensity ratio (P131/Δ*alg3*) > 2; Group 2 includes the proteins with no significant difference in abundance between P131 and Δ*alg3.* Group 3 included the proteins that were not detected in P131 or the proteins showing a significantly lower abundance in P131 with intensity ratio (P131/Δ*alg3*) < 0.5. Student’s *t*-test was used for statistical analysis and abundance difference was considered significant with *P* < 0.05. LFQ, lable-free quantification; TMHMM, Transmembrane Hidden Markov Model.

**Table 3:** **List of the51putative Alg3-regulated secretory proteins**

**Protein annotation**	**Protein accession No.**	**No. of unique peptides**	**Sequence coverage** **(%)**	**MW** **(kDa)**	**Average LFQ intensity**		**Intensity ratio (P131/Δ*alg3*)**	***P* value**
					**P131**	Δ***alg3***		
Invertase 1	MGG_05785	18	34.6	71.804	1.48E+10	0	+∞	NA
Endoglucanase C	MGG_01956	9	24.8	36.915	1.73E+08	0	+∞	NA
Hypothetical protein	MGG_00210	5	30.7	29.468	1.72E+08	0	+∞	NA
Protease inhibitor	MGG_08772	4	22.2	31.713	1.19E+08	0	+∞	NA
Carbohydrate esterase	MGG_12016	4	27.5	25.041	1.47E+08	0	+∞	NA
Cell wall protein	MGG_09460	3	16.7	25.663	1.09E+08	0	+∞	NA
Endothiapepsin	MGG_13598	5	13.4	48.017	8.95E+07	0	+∞	NA
Cell wall mannoprotein	MGG_05092	4	19	39.375	1.00E+08	0	+∞	NA
Mitochondrial iron uptake protein	MGG_02435	4	30.2	21.905	6.09E+07	0	+∞	NA
Galactose oxidase	MGG_03826	4	16.3	37.936	4.40E+07	0	+∞	NA
Hypothetical protein	MGG_06523	5	30.6	24.388	2.79E+07	0	+∞	NA
Serine-threonine rich protein	MGG_01009	2	20.3	15.298	3.25E+07	0	+∞	NA
HSP70-like protein	MGG_03983	4	25.3	23.384	2.09E+07	0	+∞	NA
1,2-dihydroxy-3-keto-5-methylthiopentene dioxygenase 2	MGG_06443	4	27.7	20.661	1.90E+07	0	+∞	NA
Cellobiose dehydrogenase	MGG_07949	2	13	30.51	1.04E+08	0	+∞	NA
α-amylase 3	MGG_10209	6	18	61.346	7.99E+07	0	+∞	NA
Surface protein 1	MGG_07791	2	18.7	13.953	1.09E+08	0	+∞	NA
Hypothetical protein	MGG_08971	4	41.1	20.736	9.22E+09	1.16E+08	79.21	0.029
DJ-1 family protein	MGG_10466	5	21.1	28.217	2.50E+09	3.86E+07	64.91	0.009
Phytase	MGG_06167	10	25.9	57.625	1.51E+09	1.03E+08	14.68	0.043
Hypothetical protein	MGG_08655	8	40.8	42.037	5.12E+09	7.09E+08	7.21	0.002
Ligninase H2	MGG_07790	15	42.2	51.333	1.94E+09	3.01E+08	6.45	0.014
1,3-β-glucanosyltransferase gel3	MGG_08370	10	27.4	55.871	4.46E+08	6.96E+07	6.41	0.004
Ecm33	MGG_06189	12	32.8	40.639	4.86E+09	8.56E+08	5.68	0.000
Gdsl-like lipase acylhydrolase	MGG_05879	3	19.8	26.702	4.28E+07	7.72E+06	5.54	0.035
Peptidase a1 protein	MGG_09818	11	44.5	48.357	2.93E+09	5.49E+08	5.34	0.034
Eukaryotic aspartyl protease	MGG_03014	5	13.1	53.58	1.62E+08	3.06E+07	5.30	0.013
Endoglucanase C	MGG_13993	7	26.2	33.254	1.17E+09	2.34E+08	4.99	0.011
Cell wall glucanosyltransferase	MGG_00592	16	54	38.442	7.25E+09	1.62E+09	4.47	0.000
FAD dependent oxidoreductase	MGG_02295	10	28.5	50.565	5.89E+08	1.35E+08	4.38	0.033
Acidic mammalian chitinase	MGG_04732	15	39.3	45.789	3.80E+09	8.72E+08	4.37	0.042
Hypothetical protein	MGG_00283	7	56.8	16.073	5.32E+10	1.32E+10	4.04	0.025
Acid phosphatase	MGG_03439	17	68.3	31.695	5.56E+09	1.43E+09	3.90	0.040
Acetylxylan esterase 2	MGG_09840	8	27.8	30.582	2.65E+09	6.99E+08	3.80	0.000
Hypothetical protein	MGG_08275	4	29.2	21.252	1.99E+08	5.36E+07	3.71	0.047
ThiJ/PfpI family protein	MGG_01085	8	51.4	26.67	3.39E+10	9.15E+09	3.70	0.046
Hypothetical protein	MGG_04944	8	39.3	27.294	3.12E+09	8.70E+08	3.59	0.018
Chitinase	MGG_17153	13	40.2	46.36	5.92E+09	1.72E+09	3.45	0.003
Phosphorylcholine phosphatase	MGG_08342	6	24.4	39.875	1.95E+08	5.76E+07	3.38	0.017
Acid phosphatase	MGG_00552	8	25.9	50.763	3.57E+08	1.08E+08	3.31	0.013
Septation protein SUN4	MGG_00505	10	38.2	43.597	2.28E+10	7.78E+09	2.93	0.028
Cell wall glucanase	MGG_01134	4	14.6	46.89	7.15E+07	2.51E+07	2.85	0.047
Lipocalin-like domain-containing protein	MGG_03369	3	23	20.849	7.53E+07	2.65E+07	2.85	0.028
Calcineurin-like phosphoesterase	MGG_05689	9	33.8	49.561	3.47E+08	1.29E+08	2.68	0.000
Glycoside hydrolase	MGG_04582	9	22.6	55.891	1.71E+09	6.43E+08	2.66	0.034
Carboxypeptidase S1	MGG_03995	4	18.4	63.027	1.15E+08	4.44E+07	2.59	0.050
Hypothetical protein	MGG_09321	12	57.7	38.315	9.25E+09	3.58E+09	2.58	0.044
Adhesin protein Mad1	MGG_12523	7	13.7	72.224	1.42E+09	6.08E+08	2.33	0.022
1,3-β-glucanosyltransferase gel4	MGG_11861	14	29.7	56.562	7.08E+09	3.17E+09	2.23	0.011
Endothelin-converting enzyme	MGG_06643	31	47.8	85.844	3.65E+09	1.67E+09	2.19	0.012
Glucan 1,3-β-glucosidase	MGG_04689	11	42.1	42.225	3.17E+09	1.50E+09	2.11	0.008

*Note*: The 51 proteins in Group 1 as indicated in Table 2 were identified as potential Alg3-regulated secretory proteins, including proteins that were not detected in Δ*alg3* or the proteins showing a significantly higher abundance in P131 with intensity ratio (P131/Δ*alg3*) > 2 (Student’s *t*-test; *P* < 0.05). No. of unique peptides refers to the total number of different peptides that were assigned to the same protein. NA, not applicable.

We used SignalP (http://www.cbs.dtu.dk/services/SignalP-0.4.1/), SecretomeP (http://www.cbs.dtu.dk/services/SecretomeP/), and Transmembrane Hidden Markov Model (TMHMM, http://www.cbs.dtu.dk/services/TMHMM/) to validate the secretory nature of the proteins identified from the secretomes. SignalP predicts the N-terminal signal peptides for proteins secreted through the ER-Golgi pathway, SecretomeP predicts non-classical secreted proteins, and TMHMM predicts possible transmembrane domains for membrane-anchored proteins. For Group 1 proteins, 43 proteins were predicted to contain a signal peptide (SP), 7 proteins were predicted by SecretomeP, and 11 proteins were predicted to have one TM domain. Therefore, 62.7% of Group 1 proteins are secreted proteins, and 21.6% are membrane proteins. Following the same calculation, 35.9% of Group 2 proteins are secreted proteins, and 7.5% are membrane proteins; only 9.0% of Group 3 proteins are secreted proteins, and 6.7% are membrane proteins. From the Group 3 proteins that were specifically detected with higher abundances in the secretome of Δ*alg3* strain, a much smaller proportion are secreted proteins, suggesting that many non-secreted proteins are abnormally secreted in the Δ*alg3* strain, possibly because the cell wall integrity is impaired in Δ*alg3* mutant; however, those proteins are not of interest in this study (Table 2, Table S2).

**Figure 2:**
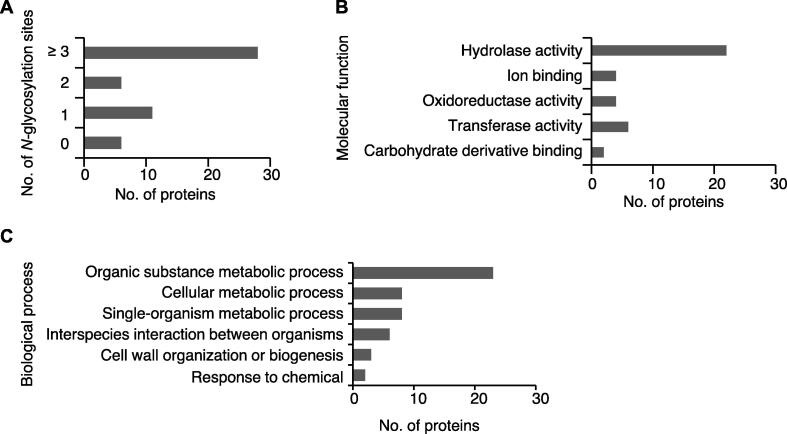
**Bioinformatic analysis of secreted proteins requiring Alg3 for proper secretion** **A.** Distribution of *N*-glycosylation sites in the secreted proteins requiring Alg3 for proper secretion predicted by NetNGlyc analysis. **B.** GO enrichment analysis of the 51 putative Alg3-regulated secreted proteins classified with molecular function terms. **C.** GO enrichment analysis of the 51 putative Alg3-regulated secreted proteins classified with biological process terms. GO, Gene Ontology.

In further analysis, we focus on the Group 1 proteins listed in Table 3, since those 51 proteins are putative Alg3-regulated proteins. We used *N*-GlycoSite analysis (http://www.cbs.dtu.dk/services/NetNGlyc/) to predict the possible *N*-linked glycosylation sites for them. Among them, 28 proteins (55%) have more than two predicted *N*-glycosylation sites, while 6 (12%), 11 (21%), and 6 (12%) proteins contain two, one, or no predicted *N*-glycosylation sites, respectively (Figure 2A; Table S2). Thus, most proteins regulated by Alg3 contain predicted *N*-glycosylation sites.

Gene Ontology (GO) enrichment analysis was performed for the 51 putative Alg3-regulated secretory proteins. Apart from eight hypothetical proteins with unknown functions, many proteins were detected with hydrolase, ion binding, and oxidoreductase activity (Figure 2B). Furthermore, many proteins were predicted to play a role in metabolic processes, *e.g.*, organic substance metabolic process, cellular metabolic process, and single-organism metabolic process (Figure 2C), suggesting that those secreted proteins strongly influence *M. oryzae* metabolic processes. Other proteins were associated with cell wall organization, response to chemicals, or interaction between organisms (Figure 2C).

### Alg3 affects the secretion and ***N***-glycosylation of Group 1 proteins

**Figure 3:**
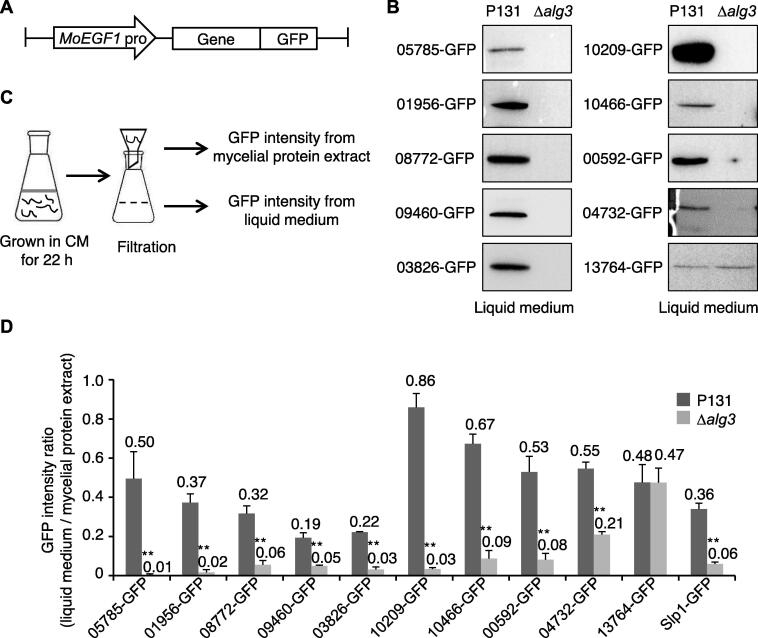
**Secretion of the candidate Alg3-regulated proteins** **A.** Schematic diagram of constructed vectors for tested genes. *MoEGF1* pro, *M. oryzae elongation factor 1* promoter; Gene, genomic DNA sequence of a target gene; GFP, green fluorescence protein. Each constructed vector was transformed into wild-type P131 and Δ*alg3* strains, respectively. **B.** Immunoblotting analysis of tested proteins from liquid media of wild-type P131 and Δ*alg3* strains with anti-GFP antibody. Three replicates were performed for each strain, and one set of representative images was shown. **C.** Experimental strategy to measure the GFP intensity from mycelial protein extracts and CM liquid media. The GFP intensity ratio was calculated as the GFP intensity from 100 μl liquid medium against that from 0.1 g mycelial protein extract. **D.** The GFP intensity ratio for the tested secreted proteins in wild-type P131 and Δ*alg3* strains. Slp1, a known effector protein regulated by Alg3, was used as a positive control. Error bars denote standard deviations from three biological replicates. **, significant differences between the wild-type and mutants (*P* < 0.01, Student’s *t*-test).

To further confirm the secretion of the identified proteins specifically regulated by Alg3, proteins were expressed in the *M. oryzae* P131 strain and Δ*alg3* strain, respectively, with a C-terminal GFP tag, under the control of the promotor from a constitutively expressed *M. oryzae* gene *MoEGF1* [14] (Figure 3A). The following proteins were selected for the validation tests: 1) Group 1 proteins MGG_05785, MGG_01956, MGG_08772, MGG_09460, MGG_03826, MGG_10209, MGG_10466, MGG_00592, and MGG_04732, 2) Group 2 protein MGG_13764, and 3) Group 3 proteins MGG_03670, MGG_10234, and MGG_10318 (Table 2, Table S2).

We cultured various fungal strains in liquid CM and detected the levels of proteins in the secreted CM using an anti-GFP antibody. We found that 05785-GFP, 01956-GFP, 08772-GFP, 09460-GFP, 03826-GFP, 10209-GFP, and 10466-GFP were detected in the CM of P131 strain but not of Δ*alg3* strain, 00592-GFP and 04732-GFP were detected in the CM of P131 strain and barely detectable in that of Δ*alg3* strain, and 13764-GFP was detected in the CM of both P131 and Δ*alg3* strains (Figure 3B). However, 03060-GFP, 10234-GFP, and 10318-GFP were undetectable in the CM of P131 and Δ*alg3* strains (Figure S2A). The immunoblotting assay confirmed our secretome data that these Group 1 proteins require Alg3 for proper secretion.

We developed another method to directly test protein secretion in the liquid medium without a protein extraction step. The fluorescence signal from a functional GFP fusion can be easily measured using a microplate reader, and the fluorescence intensity correlates to the protein expression level (Figure 3C). Therefore, the liquid CM was loaded in the microplate reader, and the fluorescence intensity was measured to detect secreted GFP fusion proteins. Considering that the same vector might integrate into different genomic regions in different strains, we could not directly compare the measured fluorescence intensity between P131 and Δ*alg3* strains harboring the same GFP fusion protein. Therefore, the fluorescence intensity of protein extracts from a certain weight (0.1 g) of *M. oryzae* mycelia was also measured for normalization. For each strain, the fluorescence signals of the liquid CM were divided by that of mycelium protein extracts and calculated as a GFP intensity ratio to determine the level of secretion of each protein (Figure 3C). To test whether this measurement works for secreted proteins, the fluorescence intensity of the P131 strain expressing control GFP alone was measured, and the rate of secretion was calculated as 0.08, indicating that unfused GFP was not secreted into the liquid CM. Moreover, Slp1-GFP was secreted in the P131 strain at a rate of 0.36, while the nuclei-localized protein MoGrp1-GFP was secreted at a rate of 0.06, supporting the observation that Slp1-GFP but not MoGrp1-GFP was secreted into the liquid CM in the P131 strain [29] (Figure S2B). Furthermore, in the Δ*alg3* strain, Slp1-GFP was secreted at a rate of 0.06, confirming the finding that the secretion of Slp1-GFP is impaired in the Δ*alg3* strain. These controls confirm that this measurement and calculation method is suitable to detect secreted GFP fusion proteins in *M. oryzae* strains.

Using the fluorescence detection method described above, we validated that proteins MGG_05785, MGG_01956, MGG_08772, MGG_09460, MGG_03826, MGG_10209, MGG_10466, and MGG_00592 were secreted in the liquid CM from the P131 strains but were not secreted from the Δ*alg3* strains. Protein MGG_04732 was secreted in the liquid CM of the P131 strains but with a reduced level in the liquid CM of Δ*alg3* strains (Figure 3D), indicating that these Group 1 proteins were indeed secreted proteins regulated by Alg3. The control protein MGG_13764 was secreted in the liquid CM of both P131 and Δ*alg3* strains (Figure 3D), which further confirmed our secretome data on Group 2 proteins.

**Figure 4:**
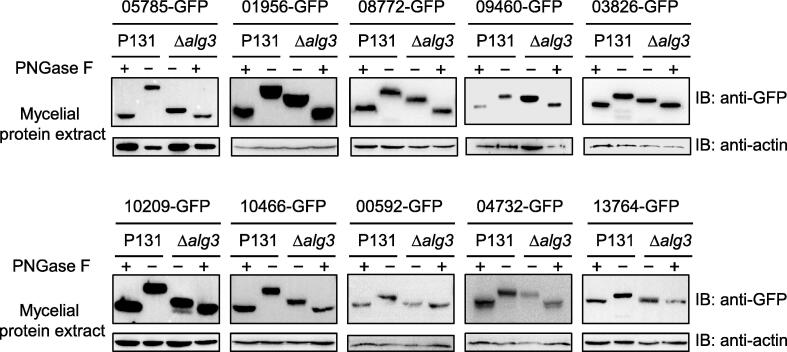
**Alg3 affects the *N*-glycosylation of candidate secreted proteins** Mycelial protein lysates with (+) or without (−) PNGase F treatment were immunoblotted with an anti-GFP antibody. The protein loading control was detected using an anti-actin antibody. PNGase F, peptide *N*-glycosidase F.

We next tested whether those secretory proteins contain the *N*-glycosylation modification. Our previous study demonstrated that the client protein Slp1 was *N*-glycosylated with Man_5_GlcNAc_2_ (instead of normal Man_9_GlcNAc_2_) in the Δ*alg3* mutants, and the size difference was observed using immunoblotting analysis [28]. We found that all the tested proteins differed in size between the P131 and Δ*alg3* strains (Figure 4), indicating that complete *N*-glycosylation of the aforementioned proteins occurs in the P131 strain, while only partial *N*-glycosylation takes place in the Δ*alg3* strain. We also applied the peptide *N*-glycosidase F (PNGase F) [30], an amidase that cuts the NAcGlc group and the Asn residues from *N*-glycosylated proteins, and detected the de-glycosylated form of each protein in the P131 strain (Figure 4). The different protein bands between the minus and plus PNGase F treatments in the Δ*alg3* strains suggested that the partial *N*-glycosylation of glycoproteins was also cleaved by PNGase F. Our findings reveal that the identified secretory proteins contain the *N*-glycosylation modification and depend on Alg3 for a complete *N*-glycosylation.

**Figure 5:**
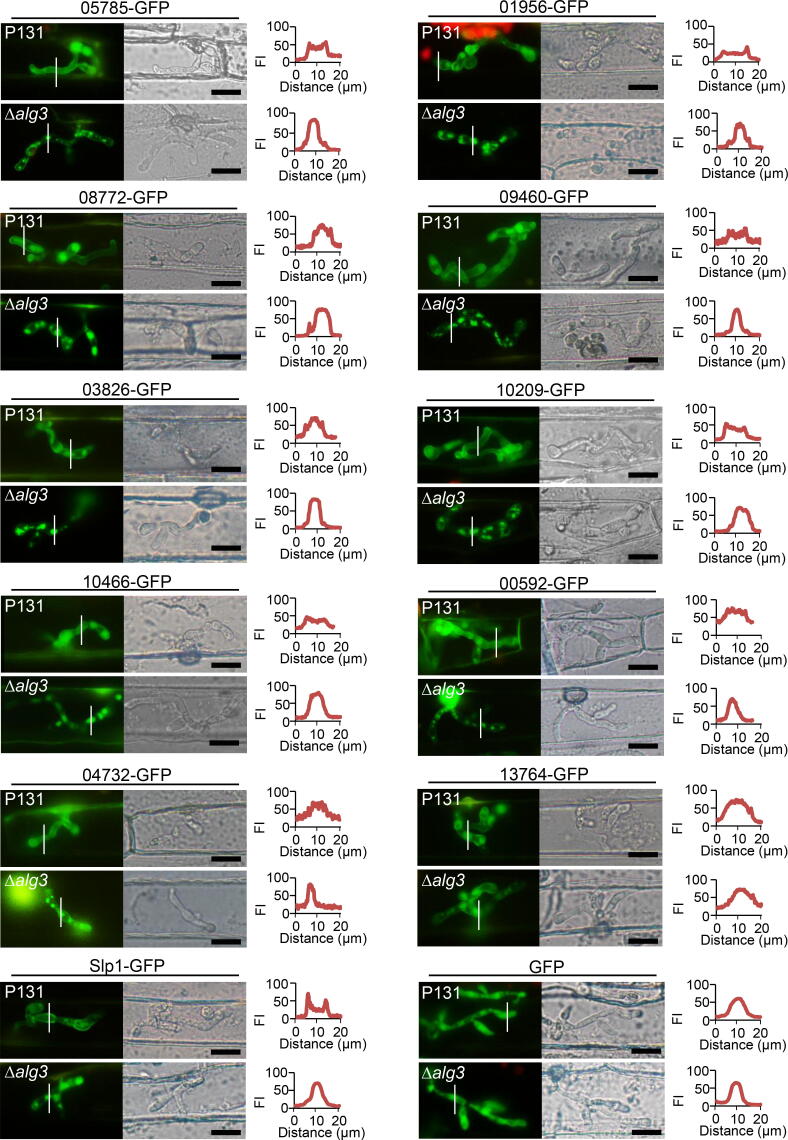
**Alg3 affects thesubcellularlocalization of candidate secreted proteins** Left panels, fluorescence signals imaged in the infection hyphae growing in barley epidermal cells for the tested proteins. White lines in the fluorescence images show the path for FI measurement. Middle panels, bright-field images for the infection hyphae. Right panels, FI in transverse sections of the infection hyphae along the white line. Slp1-GFP and GFP alone were used as the positive and negative controls, respectively, at the bottom. One representative picture out of 5 independent infection hyphae is shown for each strain. FI, fluorescence intensity. Scale bar, 20 μm.

Since those secretory proteins were identified from the liquid medium during fungal vegetative growth, we wondered whether they also localized in the infection hypha during the plant–fungus interaction. We investigated their subcellular localization in the infection hyphae using a fluorescence microscope. In P131 strains transformed with GFP-labeled MGG_05785, MGG_01956, MGG_08772, MGG_09460, MGG_03826, MGG_10209, MGG_10466, MGG_00592, MGG_04732, and MGG_13764, the GFP signals were distributed in the cytoplasm and at the plant–fungus interface (Figure 5), similar to Slp1-GFP in the P131 strain. In Δ*alg3* strains transformed with GFP-labeled MGG_05785, MGG_01956, MGG_08772, MGG_09460, MGG_03826, MGG_10209, MGG_10466, MGG_00592, and MGG_04732, the GFP signals were restricted in small dot-like structures within the infection hyphae, and only a small portion of GFP signals were located at the plant–fungus interface (Figure 5), indicating that *N*-glycosylation is important for the subcellular localization of those proteins. In Δ*alg3* strains expressing MGG_13764-GFP, the GFP signals were similar to that in P131 strains, indicating that *N*-glycosylation modification is not essential for the subcellular localization of MGG_13764 protein.

### MGG_05785 is important for fungal pathogenicity and cell wall integrity

**Figure 6:**
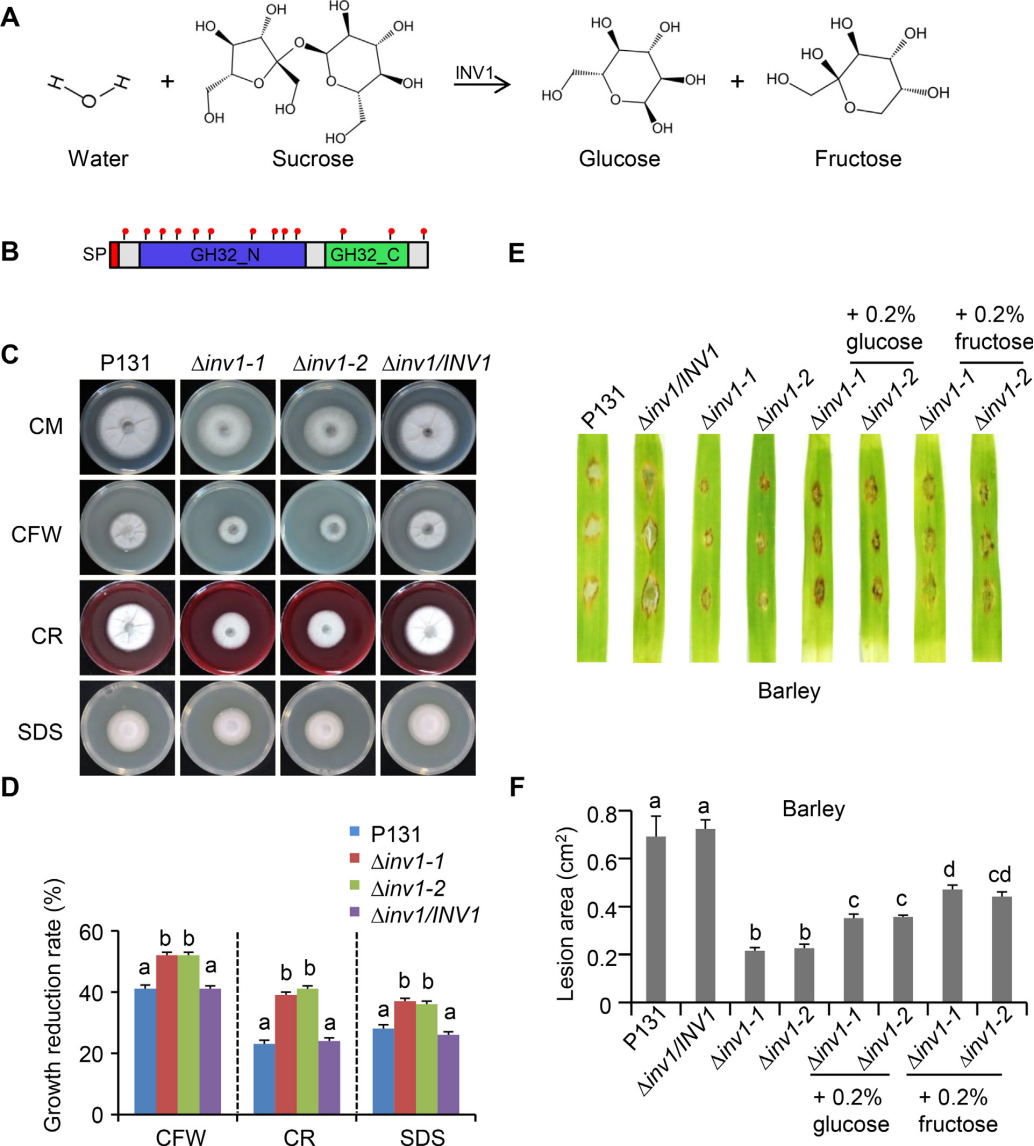
**MGG_05785 is important for fungal virulence and cell wall integrity** **A.** Schematic diagram showing that MGG_05785/INV1 functions in the decomposition of sucrose. **B.** Deduced protein domains of INV1. Red dots indicate the predicted *N*-glycosylation sites. **C.** Colony growth of strains P131, Δ*inv1-1*, Δ*inv1-2*, and Δ*inv1/INV1* on CM supplemented with different cell wall-perturbing agents, including 0.1 mg/ml CFW, 0.2 mg/ml CR, and 0.005% SDS. The cultures were incubated at 28 °C for 5 days before being photographed. **D.** Quantification of the growth reduction rates of mycelia growth on CM supplemented with different cell wall-perturbing agents. Growth reduction rate (%) = (diameter on normal CM − diameter on CM with chemical)/diameter on normal CM. The letters indicate significantly different groups (*P* < 0.001, one-way ANOVA with post-hoc Turkey tests) for corresponding strains. **E.** Barley drop-inoculation assay. Barley leaves drop-inoculated with conidial suspensions (2 × 10^4^ spores/ml) of the strains P131, Δ*inv1*, and Δ*inv1/INV1* without or with exogenous glucose or fructose were photographed at 5 dpi, and the absolute lesion area was calculated. **F.** Quantification of lesion area in the barley drop-inoculation assay as shown in (E). Error bars denote standard deviations from three biological replicates. The letters indicate significantly different groups (*P* < 0.05, one-way ANOVA with post-hoc Turkey tests) for corresponding strains or treatments. SP, singal peptide; GH32_N, GH32 N-terminal domain; GH32_C, GH32 C-terminal domain; CFW, calcofluor white; CR, Congo red; dpi, days post inoculation.

From the LC–MS/MS data, INV1, encoded by *MGG_05785*, is the most abundant protein identified only in liquid CM of the P131 strain (Table 3). The *N*-glycosylation modification of INV1 was confirmed in the mycelium proteins of *M. oryzae* strain P131 expressing INV1-GFP fusion proteins, and this process was mediated by Alg3 (Figure 4). Invertase is a major enzyme present in plants and microorganisms [31], and it carries out the irreversible conversion of sucrose to glucose and fructose (Figure 6A). A previous study showed that *M. oryzae* Δ*inv1* mutants had reduced fitness during plant infection [32]. INV1 is a protein with 660 amino acids, containing an SP as a leading sequence, a GH32 N-terminal domain, and a GH32 C-terminal domain (Figure 6B). It contains 13 predicted *N*-glycosylation sites dispersed among the different domains (Figure 6B).

We further characterized the function of INV1 in cell wall integrity and virulence by generating two independent knockout mutants in the P131 background, Δ*inv1-1* and Δ*inv1-2*; both mutants were confirmed with Southern blot analysis (Figure S3A and B). Later on, we generated the complementation strain by transforming *INV1* genomic sequence with a C-terminal GFP tag into Δ*inv1-1* mutant, and the resulting Δ*inv1/INV1* strain could restore the mutant phenotype. The Δ*inv1* mutant strains grew normally on oatmeal-tomato agar (OTA) plates (Figure S3C). To confirm its invertase function, the Δ*inv1* mutant strains were grown on solid MM without a carbon source or were supplied with sucrose, glucose, or fructose as the only carbon source. Since the size of the colonies was similar among the P131 strain and Δ*inv1* mutant strains, the thickness of the fungal growth reflects the carbon absorption efficiency. The mycelium of the P131 strain was very thin on the solid MM without a carbon source but was much thicker on the solid MM with sucrose, glucose, or fructose. By contrast, the mycelium of the Δ*inv1* mutant strains was thicker only on the solid MM with glucose or fructose but not with sucrose, confirming that the Δ*inv1* mutants could not utilize sucrose for fungal mycelium growth (Figure S3D). The Δ*inv1/INV1* complementation strain restored the sucrose utilization defect of the Δ*inv1-1* mutant, indicating that the expressed INV1-GFP fusion protein is functional (Figure S3D). The fungal mycelial weights of Δ*inv1* mutant strains were also measured in the liquid MM supplied with different carbon sources, and the same results were observed (Figure S3E). Therefore, we concluded that INV1 plays an important role in sucrose utilization.

In our previous report, we observed defects of cell wall integrity in the Δ*alg3* strains but not in the Δ*slp1* mutant [28], suggesting that other secretory proteins regulated by Alg3 should function in cell wall integrity. Since INV1 is the most abundant protein secreted in CM during mycelium growth, we wondered whether INV1 plays a role in cell wall integrity. We grew Δ*inv1* mutant strains on common cell wall-perturbing agents, including calcofluor white (CFW), Congo red (CR), and SDS [28]. When the P131 strain and the complementation strain Δ*inv1/INV1* were grown on CM with those agents, the vegetative growth was reduced by 41% with CFW, 23% with CR, and 28% with SDS, in comparison with their growth on CM. By contrast, the growth of Δ*inv1-1* and Δ*inv1-2* was reduced by 52% with CFW, 39% with CR, and 37% with SDS, suggesting a significantly increased sensitivity to all three tested cell wall-perturbing agents (*P* < 0.001, one-way ANOVA with post-hoc Turkey tests) (Figure 6C and D). We noticed that the sugar source commonly used in CM is sucrose, which cannot be utilized by the Δ*inv1* mutants. When we replaced sucrose with glucose in this experiment, we found that all strains had similar reductions in growth rates on plates with CFW, CR, and SDS (Figure S3F and G). The growth assays in CM with different cell wall-perturbing agents revealed that fungal cell wall integrity is associated with normal carbon utilization in Δ*inv1* mutants.

We questioned whether the reduced pathogenicity of Δ*inv1* mutants was also associated with carbon uptake. Therefore, we investigated the role of INV1 in *M. oryzae* pathogenicity. Consistent with the previous report [32], the Δ*inv1* mutants showed significantly reduced virulence on rice and barley (*Hordeum vulgare*) leaves (*P* < 0.01, one-way ANOVA with post-hoc Turkey tests) (Figure S3H–K). Furthermore, we added an additional carbon source to see whether it could restore the mutant’s virulence. In drop-inoculation assay, the symptom lesions from Δ*inv1* strains were much narrower than from the P131 strains on barley leaves (Figure 6E). When 0.2% glucose or 0.2% fructose was applied in the drop-inoculation assay, the pathogenicity of Δ*inv1* mutants was partially restored, although not to the level of P131 strains (Figure 6E and F). Therefore, carbon utilization is partially linked to the fungal virulence in Δ*inv1* mutants.

### MGG_04732 is important for fungal pathogenicity and cell wall integrity

**Figure 7:**
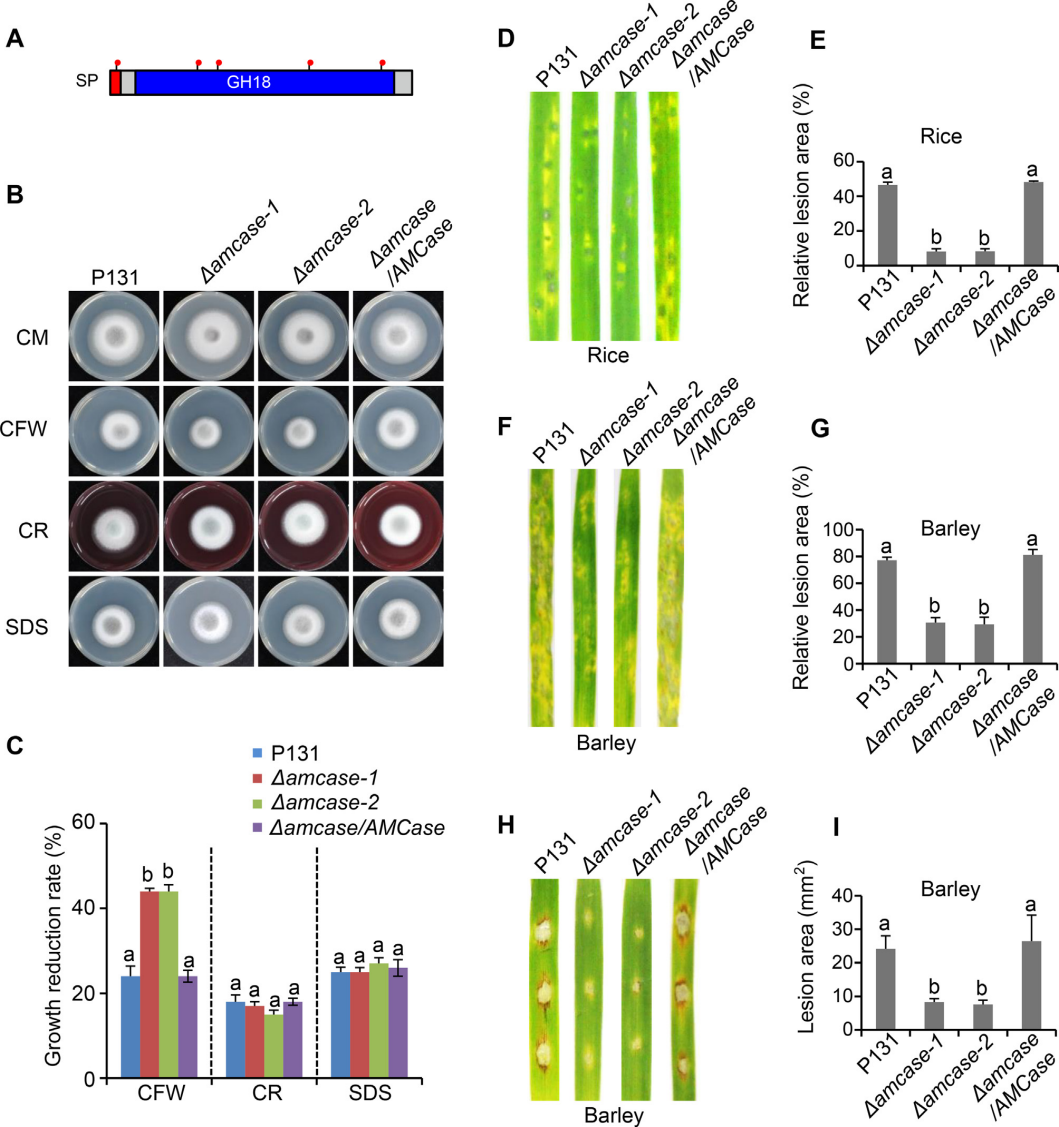
**MGG_04732 is important for fungal virulence and cell wall integrity** **A.** Deduced protein domains of MGG_04732/AMCase. Red dots indicate the predicted *N*-glycosylation sites. **B.** Colony growth of strains P131, Δ*amcase-1*, Δ*amcase-2*, and Δ*amcase/AMCase* on CM supplemented with different cell wall-perturbing agents, including 0.1 mg/ml CFW, 0.2 mg/ml CR, and 0.005% SDS. The cultures were incubated at 28 °C for 5 days before being photographed. **C.** Quantification of the growth reduction rates of mycelia growth on CM supplemented with different cell wall-perturbing agents. The letters indicate significantly different groups (*P* < 0.001, one-way ANOVA with post-hoc Turkey tests) for corresponding strains in the growth assays. **D.** Rice spraying assay. Rice leaves sprayed with conidial suspensions (1 × 10^5^ spores/ml) of the indicated strains were photographed at 5 dpi, and the relative lesion area was calculated. **E.** Quantification of relative lesion area in rice spraying assay as shown in (D). **F.** Barley spraying assay. Barley leaves sprayed with conidial suspensions (1 × 10^4^ spores/ml) of the indicated strains were photographed at 5 dpi, and the relative lesion area was calculated. **G**. Quantification of relative lesion area in barley spraying assay as shown in (F). **H.** Barley drop-inoculation assay. Barley leaves drop-inoculated with conidial suspensions (5 × 10^4^ spores/ml) of the indicated strains were photographed at 5 dpi, and the absolute lesion area was calculated. **I.** Quantification of lesion area in the barley drop-inoculation assay as shown in (H). Error bars denote standard deviations from three biological replicates. The letters indicate significantly different groups (*P* < 0.01, one-way ANOVA with post-hoc Turkey tests) for corresponding strains in the infection assays.

We next tested the gene *MGG_04732*, encoding a protein with homolog to AMCase containing an SP and a glycosyl hydrolase family 18 (GH18) domain, with 5 predicted *N*-glycosylation sites (Figure 7A). The *N*-glycosylation of AMCase was confirmed in the mycelial protein extracts of P131, and this process was mediated by Alg3 (Figure 4). *M. oryzae* genome has 15 genes encoding GH18 family chitinases [33], [34], among which only *Chia1* was characterized to be important for fungal pathogenicity [35]; the function of *AMCase* was not reported.

We wondered whether AMCase plays a role in cell wall integrity and pathogenicity; two independent knockout mutants Δ*amcase-1* and Δ*amcase-2* were generated and confirmed by Southern blot analysis (Figure S4A and B). We also generated the complementation strain by transforming *AMCase* genomic sequence with a C-terminal GFP tag into Δ*amcase-1* mutant, and the resulting Δ*amcase/AMCase* strain could restore the mutant phenotype. Those knockout mutant strains grew and produced spores normally on the OTA plates (Figure S4C–E). In the cell wall integration test, when the P131 strain was grown on CM with cell wall-perturbing agents, vegetative growth was reduced by 24% with CFW, 18% with CR, and 25% with SDS, in comparison with their growth on CM. By contrast, the growth of Δ*amcase-1* and Δ*amcase-2* was reduced by 44% with CFW, 17% with CR, and 25% with SDS, suggesting that the mutants had a significantly increased sensitivity only to CFW (*P* < 0.001, one-way ANOVA with post-hoc Turkey tests), but not to CR and SDS (Figure 7B and C). The Δ*amcase/AMCase* complementation strain restored the cell wall integrity defect of the Δ*amcase-1* mutant, indicating that the expressed AMCase-GFP fusion protein is functional. To test whether AMCase might function in exogenous chitin utilization, we grew the wild-type P131 and knockout strains on the solid MM without carbon source or supplied with glucose and chitin as the sole carbon sources. However, we could not observe the growth difference between P131 and Δ*amcase* mutants under those conditions (Figure S4F and G), suggesting that AMCase could not decompose exogenous chitin. In the pathogenicity assay, we did spray infection on rice and barley plants and drop infection on barley plants using conidiospore solution. The two Δ*amcase* mutants showed significantly reduced pathogenicity on rice and barley leaves (*P* < 0.01, one-way ANOVA with post-hoc Turkey tests; Figure 7D–I), suggesting that AMCase plays an important role in fungal pathogenicity.

### *N*-glycosylation is essential for the proper function of AMCase

**Figure 8:**
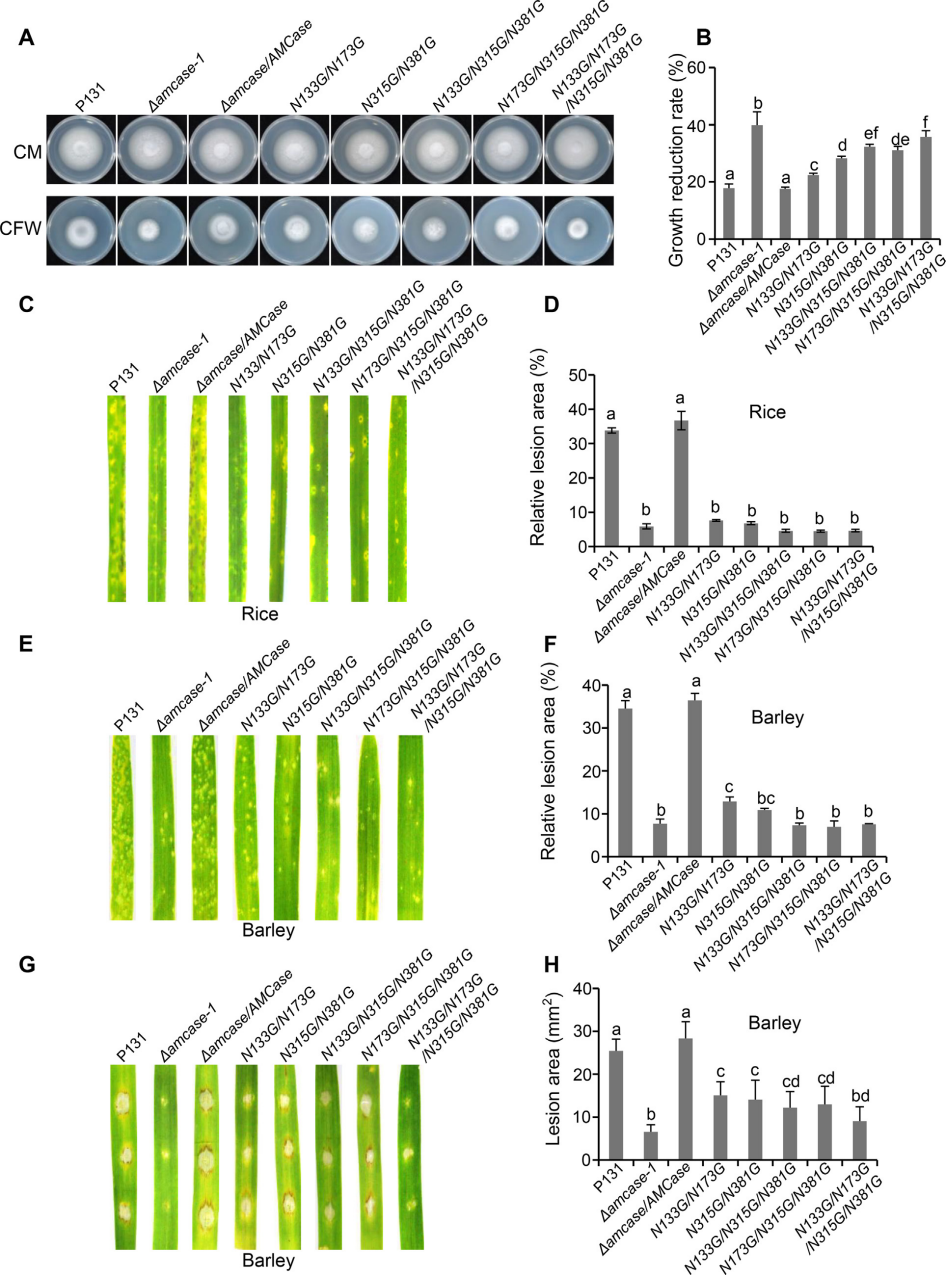
***N*-glycosylation is essential for the function of AMCase** **A.** Colony growth of strain P131, Δ*amcase-1*, Δ*amcase/AMCase*, *N133G/N173G*, *N315G/N381G*, *N133G/N315G/N381G*, *N173G/N315G/N381G*, and *N133G/N173G/N315G/N381G* on CM supplemented with 0.1 mg/ml CFW. The cultures were incubated at 28 °C for 5 days before being photographed. **B.** Quantification of the growth reduction rates of mycelia growth on CM supplemented with CFW as shown in (A). **C.** Rice spraying assay. Rice leaves sprayed with conidial suspensions (1 × 10^5^ spores/ml) of the indicated strains were photographed at 5 dpi, and the relative lesion area was calculated. **D.** Quantification of relative lesion area in rice spraying assay as shown in (C). **E.** Barley spraying assay. Barley leaves sprayed with conidial suspensions (1 × 10^4^ spores/ml) of the indicated strains were photographed at 5 dpi, and the relative lesion area was calculated. **F.** Quantification of relative lesion area in barley spraying assay as shown in (E). **G.** Barley drop-inoculation assay. Barley leaves drop-inoculated with conidial suspensions (5 × 10^4^ spores/ml) of the indicated strains were photographed at 5 dpi, and the absolute lesion area was calculated. **H.** Quantification of lesion area in the barley drop-inoculation assay as shown in (G). Error bars denote standard deviations from three biological replicates. The letters indicate significantly different groups (*P* < 0.05, one-way ANOVA with post-hoc Turkey tests) for the tested fungal strains.

To verify the role of *N*-glycosylation for proper function of AMCase, the *AMCase* genomic sequences with different point mutations and a C-terminal GFP tag were generated and transformed into the Δ*amcase-1* mutant strain. Among the 5 predicted *N*-glycosylation sites of AMCase, N5, which is located in the SP region, was not tested; N133, N173, N315, and N381 located in the annotated GH18 domain were mutated in functional tests. Single mutant strains N133G, N173G, N315G, and N381G grew similarly to wild-type strain P131 in response to CFW (Figure S5A and B) and formed disease lesions in rice and barley plants almost to the level of P131 strain (Figure S5C–H). In the meanwhile, the GFP signals from the liquid cultures of those single mutant strains were also detected at a level similar to the Δ*amcase/AMCase* complementation strain (Figure S6A), indicating that single point mutations do not affect much the function and the secretion of AMCase. We then generated a series of double, triple, quadruple mutant strains for further tests. The double-mutants N133G/N173G and N315G/N381G, triple-mutants N133G/N315G/N381G and N173G/N315G/N381G, and the quadruple-mutant N133G/N173G/N315G/N381G were much more sensitive to CFW compared to P131, with the strongest effect in the N133G/N173G/N315G/N381G strain (*P* < 0.05, one-way ANOVA with post-hoc Turkey tests) (Figure 8A and B). Therefore, the additive effect could be observed for those point mutations grown in CFW conditions. Consistently, those tested mutants showed significantly reduced virulence in rice and barley plants, comparable to the virulence level of Δ*amcase-1* mutant strain in the spray infection, and with slight variation in the drop infection (*P* < 0.05, one-way ANOVA with post-hoc Turkey tests) (Figure 8C–H). In the meanwhile, the GFP signals were also strongly reduced in the liquid cultures of those mutants compared to the Δ*amcase/AMCase* complementation strain (Figure S6B). Abolishment of four *N*-glycosylation sites in AMCase strongly destroyed protein function and secretion, suggesting that *N*-glycosylation is essential for the proper function and secretion of AMCase.

## Discussion

Many studies have used liquid culture media to identify the secretomes from pathogenic microbes, *e.g.*, *Xanthomonas oryzae*, *Botrytis cinerea*, and *Staphylococcus aureus* [36], [37], [38], [39]. In the present study, we used a quantitative proteomics approach to analyze the secretome of *M. oryzae* and identified 558 proteins from the liquid culture media, including 411 proteins of the wild-type P131 strain and 539 proteins of the Δ*alg3* mutant strain. Among the 558 proteins, 190 were found in previous *M. oryzae* secretome studies (Table S2). In a previous study, 51 proteins were identified by comparative secretome analyses of *M. oryzae* grown in CM, MM, and MM lacking N (MM-N) media, of which 34 proteins (66.7%) were also identified in our dataset [40]. In another study, the secretomes of conidiospores grown in glass plates, PVDF membrane, and liquid culture media were analyzed, and 52 proteins were identified [41], of which 32 proteins (61.5%) overlapped with our secretome (Table S2). Therefore, our *in vitro* secretome analysis detected much more secreted proteins compared with the two previous studies, possibly because we used gel-free rather than gel-based proteomics. Although it is a much greater challenge, *in vivo* apoplastic fluid of rice after *M. oryzae* infection was analyzed, and a total of 441 secreted proteins were identified, of which 52 proteins were from a 2-DE gel-based approach, and 425 proteins were from a gel-free MudPIT analysis [42]. When comparing this *in vivo* secretome with our *in vitro* secretome data, 171 proteins overlapped, accounting for 38.8%–41.6% of the proteins identified in the two studies (Table S2), indicating that a portion of the pathogen infection-related proteins was expressed and secreted when grown in the liquid cultures. Interestingly, many previously characterized secretory proteins were commonly identified from our secretome datasets of the P131 strain and the Δ*alg3* strain. For example, *M. oryzae* secreted protein (MSP1, MGG_05344), extracellular matrix protein (EMP1, MGG_00527), *M. oryzae* hypersensitive response-inducing protein 1 (MoHrip1, MGG_15022), and *M. oryzae* hypersensitive response-inducing protein 2 (MoHrip2, MGG_16187) were found to be important for fungal pathogenicity [43], [44], [45]. Two secreted *M. oryzae* chitin deacetylates, MoCDA1 (MGG_14966) and MoCDA 2 (MGG_08774), were found to be dispensable for *Magnaporthe* virulence but important for fungal vegetative growth under stress conditions [46]. Notably, apart from EMP1, which contains four potential *N*-glycosylation sites, the other abovementioned secretory proteins have only one or even no potential *N*-glycosylation sites, which could be explained as that *N*-glycosylation is dispensable for secretion of those proteins; thus they were detected both from the P131 strain and the Δ*alg3* strain. Therefore, in the common list of proteins secreted from both strains, some uncharacterized secretory proteins might also function in fungal pathology or vegetative growth under different stress conditions. Further investigation of those commonly secreted proteins might identify additional virulence effectors in the future.

Protein *N*-glycosylation is a significant protein modification essential for fungal infection. Mutants of protein *N*-glycosylation showing defects in fungal virulence are reported not only for *M. oryzae* [47] but also for other plant pathogens such as *Penicilium digitatum*, *B*. *cinerea*, and *Ustilago maydis* [26], [27], [48], [49], [50], [51], [52], [53], [54]. A recent study on glycoprotein proteomes in *U*. *maydis* using the wild-type and mutant strains of glucosidase 1 (gls1), had identified several proteins with *N*-glycosylation modification [55]. Comparing the secretomes of P131 and Δ*alg3* in our study, 17 proteins were totally absent in the Δ*alg3* strain, and 34 proteins had a significantly higher abundance in P131 samples than in Δ*alg3* samples; thus, these 51 proteins were considered as secretory proteins that specifically require Alg3 for their secretion (Table 3). Since Alg3 functions in the early step of oligosaccharide synthesis for *N*-glycosylated proteins, the modified proteins should contain potential *N*-glycosylation sites. Among those 51 proteins, 45 proteins that contain at least one predicted *N*-glycosylation site should be direct targets of Alg3 (Table 3); the other 6 proteins that do not have a predicted *N*-glycosylation site could be indirect targets of Alg3, as their secretion may be influenced by other Alg3-regulated target proteins. It is worth noting that our proteomics approach identified previously reported proteins involved in cell wall integrity, such as glucan elongation protein 3 (Gel3, MGG_ 08370) and glucan elongation protein 4 (Gel4, MGG_11861) [46]. Thus, the reduced secretion levels of Gel3 and Gel4 in the Δ*alg3* mutant might account for the defects in the cell wall integrity in Δ*alg3* mutants.

A subset of secretory proteins containing one predicted *N*-glycan site (protein encoded by MGG_09460), two *N*-glycan sites (MGG_13764), and more than three *N*-glycan sites (MGG_05785/INV1, MGG_01956, MGG_08772, MGG_03826, MGG_10209, MGG_10466, MGG_00592, and MGG_04732/AMCase) were chosen for a series of validation assays. We confirmed that those proteins indeed had *N*-glycosylation modification (Figure 4). The secretion test results suggest that all proteins were secreted in the growth media. The secretion and localization of most of the tested proteins were affected in the Δ*alg3* mutant, validating that *N*-glycosylation is important for protein secretion and proper localization. Both the secretion and the localization of the protein encoded by *MGG_13764* were not affected in the Δ*alg3* mutant, suggesting that for this specific protein, *N*-glycosylation is not necessary for its secretion. The validation tests on those selected proteins indicate that our *in vitro* comparative secretome analysis is a powerful approach to identifying novel proteins that undergo *N*-glycosylation mediated by Alg3.

*M. oryzae* effectors consist of cytoplasmic effectors and apoplastic effectors [16]. Interestingly, apoplastic effectors Slp1 and BAS4 both contain predicted *N*-glycosylation sites, but none of the cytoplasmic effectors have a predicted *N*-glycosylation site. Thus, it is possible that *N*-glycosylation is a common feature of apoplastic effectors. We observed that all tested secretory proteins were localized at the plant–fungus interface surrounding the infection hyphae (Figure 5), which resembles the localization of Slp1, further supporting the idea that the *N*-glycosylation modification is important for protein secretion via the conventional secretory pathway.

*M. oryzae* is a hemibiotrophic fungus, which, similar to other biotrophic fungi, depends on the nutrients provided by host plants at its early infection stage. Sugar is one of the most abundant nutrients a pathogen can get from living plants. Indeed, invertase homologs have been reported in several obligate biotrophic pathogens. The invertase from the rust fungus *Uromyces fabae* was expressed during infection and localized in the extrahaustorial matrix membrane and was important in breaking down sucrose into D-glucose and D-fructose [56]. In addition, a report on wheat stripe rust (*Puccinia striiformis* f. sp. *tritici*) revealed that the invertase gene *PsINV* plays a role in *Pst* pathogenicity [57]. The *M. oryzae* genome contains four genes encoding invertases, which are clustered in a subclade when compared with other fungal invertases, but only INV1 was identified as an abundant protein in our secretome dataset. We found that *N*-glycosylation and the localization of INV1 were severely affected in the Δ*alg3* mutant; moreover, INV1 was also identified as a secreted protein in all the other *M. oryzae* secretome datasets. A previous report confirmed that a Δ*inv1* mutant showed impaired growth on sucrose-containing media, including impaired biomass formation and virulence [32]. In addition, we showed that the cell wall integrity was affected in Δ*inv1* mutants when grown in CM with sucrose, but this phenotype was completely restored when we replaced the sucrose with glucose. In a fungal virulence test, adding 0.2% glucose or 0.2% fructose also partially restored the pathogenicity of the Δ*inv1* mutants. Taken together, these observations show that carbon utilization is important for fungal cell wall integrity and virulence in *M. oryzae*.

Chitin, a homopolymer of 1,4-β-linked NAcGlc, is a structural component of the fungal cell wall [58]. Chitin fragments are well-characterized elicitors that induce plant immune responses in many plant species [59], [60]. Chitinases are chitin-degrading enzymes that are present in a wide range of organisms, including viruses, bacteria, fungi, insects, plants, and animals [61]. *M. oryzae* has 15 genes that are annotated as GH18 family chitinases. *M. oryzae* chitinase 1 (MoChia1) is involved in the fungal virulence through degrading chitin into small fragments, which would escape the recognition of plant immune receptors [35], [62]. Different chitinases have their specific expression in different cell types; AMCase maintains the highest expression level in appressorium among 15 chitinases, indicating that AMCase is likely to be associated with fungal infection [35]. Indeed, our study exhibited that Δ*amcase* mutants were less virulent on both rice and barley leaves, confirming that AMCase plays an important role in pathogenicity. In addition, we showed that Δ*amcase* mutants were highly sensitive to CFW, but not to CR and SDS, suggesting that AMCase plays a major role in response to CFW. Since CFW mainly binds to the chitin of the fungal cell wall, the sensitive response is consistent with the loss of this specific chitinase in Δ*amcase* mutants.

Notably, our secretome analysis did not identify the well-characterized apoplastic effector protein Slp1, which is known to undergo *N*-glycosylation mediated by Alg3. Since our research was conducted during vegetative mycelium growth, the expression of Slp1 might be lower than that in the *in vivo* infection condition. Many pathogenicity-related genes, especially effectors, have no or a low level of expression during vegetative growth and higher expression specifically during infection [63], [64]. Recent studies reported the functions of various transcription factors and epigenetic control in regulating effector expression in phytopathogenic fungi. For example, methylation of lysine 9 and/or lysine 27 of histone H3 (H3K9me3, H3K27me3) seems to be related to heterochromatin and effector gene silencing, and methylation of lysine 4 of histone H3 (H3K4me2) to euchromatin and effector gene expression [65], [66], [67], [68]. Further proteomics studies using related histone modification mutants would enhance our understanding of secreted effector proteins in *M. oryzae*.

## Materials and methods

### Fungal strains and growth conditions

*M. oryzae* wild-type strain P131 (field isolate) and the Δ*alg3* mutant strain which lacked the *α*-1,3-mannosyltransferase were used in this study. The Δ*alg3* strain was generated and verified previously [28]. *M. oryzae* strains with different transformants generated in this study are listed in Table S3. All *M. oryzae* strains were maintained on OTA media at 28 °C [69]. The liquid CM contains 6 g/l yeast extract, 3 g/l casein acid hydrolysate, 3 g/l casein enzymatic hydrolysate, and 10 g/l sucrose. And the liquid MM contains 6 g/l NaNO_3_, 0.502 g/l KCl, 0.502 g/l MgSO_4_·7H_2_O, 1.52 g/l KH_2_PO_4_, 1× trace element, 10 g/l D-glucose, 1% thiamine, and 0.05% biotin (pH 6.5). For the cell wall integrity test, 5-mm mycelial blocks of different strains were placed on CM agar with 0.1 mg/ml CFW (Sigma-Aldrich, Shanghai, China), 0.2 mg/ml CR (Sigma-Aldrich), and 0.005% SDS (Sigma-Aldrich). Growth reduction rate (%) = (diameter on normal CM – diameter on CM with chemical)/diameter on normal CM. For the carbohydrate supplement test, 5-mm mycelial blocks of different strains were placed on solid MM without or with different carbohydrate supplements at 28 °C for 5 days. For the mycelial wet-weight measurement, the 0.1 g mycelia were grown on liquid MM without or with different carbohydrate supplements at 28 °C for 1 day. For sporulation assay, conidia were harvested from 10-day-old OTA cultures and counted using hemocytometer for calculation.

### Virulence test

Rice (*Oryza sativa* cv. Lijiangxintuanheigu) seedlings at the third leaf stage and 7-day-old barley (*H*. *vulgare* cv. E9) seedlings were used for virulence test. The conidial suspensions of different *M. oryzae* strains were used for spray inoculation or drop inoculation as described previously [70]. Leaves were photographed at 5 days post inoculation (dpi), and relative lesion area or absolute lesion area on leaves was calculated. To test the effects of exogenous glucose and fructose on infection of the Δ*inv1* mutants, 0.2% glucose and 0.2% fructose were separately added onto the fungal inoculation sites at 18 h post inoculation after the drop inoculation, and subsequently, photographs and statistics of lesion area were taken at 5 days after inoculation.

### Secretome sample preparation

Secreted proteins were extracted from liquid medium. One gram of mycelia was collected and grown in liquid culture for 18 h; then the liquid medium was filtrated through Miracloth (Merck millipore, Beijing, China) and centrifuged at 12,000 r/min for 10 min. Then, 200 ml supernatants were collected and incubated with 12.5% (v/v) trichloroacetic acid (TCA) at 4 °C overnight to precipitate proteins. The pellets were collected after centrifuging for 30 min at 12,000 r/min, washed twice with 100% acetone, and dried. The protein samples were stored at −80 °C for further analyses.

### LC–MS/MS analyses and data processing

Proteins were digested using FASP method [71]. Peptides were separated with a Waters (Milford, MA) nanoAcquity nano-HPLC. Mobile phases were 0.1% formic acid in water (A) and 0.1% formic acid in acetonitrile (B). We used a linear gradient from 1% B to 35% B in 65 min. The trap column was Thermo Acclaim PepMap 100 (75 μm × 20 mm, C18, 3 μm). The analytical column was homemade (100 μm × 200 mm C18 stationary phase, Phenomenex, Aqua 3 μm 125 Å). Nanospray ESI–MS was performed on a Thermo Q Exactive high-resolution mass spectrometer (ThermoFisher Scientific, Waltham, MA) in a data-dependent mode.

Raw MS files were subjected to MaxQuant software (version 1.6.0.1) for protein identification and quantification as described before [72]. Peak list was generated by Andromeda, which is a built-in engine in MaxQuant, and searched against a *Magnaporthe oryzae* database (download from https://www.broadinstitute.org) augmented with the reversed sequence. Trypsin/P was set as the enzyme for digestion. Carbamidomethyl was set as a fixed modification, while oxidation and acetyl were set as variable modifications. The maximum of missed cleavage was set as 2. Main search peptide tolerance and MS/MS match tolerance were both set as 20 ppm. False discovery rates of peptide and protein were both set at 1%. The parameters for LFQ were as follows: minor ratio count was set to 2, minor and average numbers of neighbors were 3 and 6, respectively. *P* values were calculated via Student’s *t*-test by using Excel and further corrected with multiple hypothesis testing (Benjamini–Hochberg).

### Vector construction

To generate fluorescent proteins fused with GFP, coding regions of candidate proteins were cloned into pGTN under a *MoEGF1* promoter [14]. The resulting constructs were digested with *Eco*RI and delivered into the protoplasts of P131 and Δ*alg3* strains as previously described [70]. Media with 400 g/ml neomycin (Ameresco, Solon, OH) were used to select neomycin-resistant transformants.

To generate the gene replacement constructs, flanking sequences with 1.5 kb upstream and downstream of targeted genes (*INV1* and *AMCase*) were amplified using genomic DNA of P131. The two flanking sequences were cloned into pKOV21 [70]. The resulting constructs were linearized by *Not*I and delivered into the protoplasts of P131 to generate deletion mutants, which were confirmed by Southern blot hybridization. To generate the complemention constructs, the genomic sequences with the native promoter regions of *INV1* and *AMCase* were cloned into pGTN, respectively. In addition, different *AMCase* point mutation fragments were amplified and cloned into pGTN under its native promoter. The primers used to construct the vector are listed in Table S4.

### Fluorescence intensity and subcellular localization analyses

To analyze fluorescence intensity, the transformants of candidate proteins fused with GFP were grown in liquid CM cultured at 160 r/min for 22 h. Then GFP fluorescence intensity in 100 μl filtered liquid medium or in 0.1 g mycelium proteins were measured with a microplate reader (Molecular Devices i3x, Shanghai, China). To observe subcellular localization, the transformants of these validated proteins fused with GFP inoculated in barley leaves were observed with an epifluorescence microscope (Nikon, Toyko, Japan).

### Protein *N*-glycosylation analysis

For glycosylation analysis, total proteins were extracted from mycelia using the protein lysis buffer (20 mM Tris-HCl pH 7.5, 150 mM NaCl, 0.5 mM EDTA, 0.5% NP-40). Proteins with or without PNGase F (NEB, Ipswich, MA) digestion were mixed with the loading buffer (250 mM Tris-HCl pH 6.8, 10% SDS, 0.5% bromophenol blue, 50% glycerol, 5% β-mercaptoethanol) for Western blot analysis. Antibodies of anti-GFP (1:5000; ABclonal, Wuhan, China) and anti-actin (1:10,000; ABclonal) were used to detect the GFP fusion proteins and the actin control, respectively.

## Data availability

The proteomic data reported in this study have been deposited to the ProteomeXchange Consortium via the iProX partner repository [73] (ProteomeXchange: PXD024706), which are publicly accessible at http://proteomecentral.proteomexchange.org.

## CRediT author statement

**Ning Liu:** Methodology, Validation, Formal analysis, Investigation, Writing - original draft. **Linlu Qi:** Software, Formal analysis, Data curation, Writing - original draft. **Manna Huang:** Validation, Investigation. **Deng Chen:** Validation, Investigation. **Changfa Yin:** Software, Data curation. **Yiying Zhang:** Validation, Investigation. **Xingbin Wang:** Validation, Investigation. **Guixin Yuan:** Validation, Investigation. **Rui-Jin Wang:** Software, Data curation. **Jun Yang:** Formal analysis, Supervision. **You-Liang Peng:** Conceptualization, Formal analysis, Writing - review & editing, Funding acquisition, Project administration. **Xunli Lu:** Conceptualization, Methodology, Formal analysis, Investigation, Writing - original draft, Writing - review & editing, Project administration. All authors have read and approved the final manuscript.

## Competing interests

The authors declare that they have no conflict of interests.

## Supplementary material

Supplementary Figure S1 **Secreted proteins from *M. oryzae* P131 and *Δalg3* strains A.** Western blot shows the secretion of Slp1-GFP from P131 in CM and MM liquid medium; CM, nutrient-sufficient complete medium; MM, nutrient-deficient minimal medium. Coomassie blue stained gels were used as a loading control. **B.** Silver staining of secreted protein samples from the CM liquid culture of P131 and *Δalg3* strains. Three replicates were performed for each strain.

Supplementary Figure S2 **The tests for secreted proteins fused with a GFP tag A.** Immunoblotting analysis of protein 03670-GFP, 10234-GFP, and 10318-GFP (group 3) from liquid medium and mycelial lysates from wild-type P131 and *Δalg3* strains. **B.** GFP fluorescent signal was measured in 96-well plates (upper panel), and the ratio of GFP intensity from CM liquid culture against that from mycelial protein extracts was quantified (lower panel) for Slp1-GFP, MoGrp1-GFP, control GFP samples, and background control P131 samples. Error bars denote standard deviations from three biological replicates. The letters indicate significantly different groups (*P* < 0.001, one-way ANOVA with post-hoc Turkey tests) for the tested fungal strains.

Supplementary Figure S3 **The characterization of *M. oryzae INV1* function A.** Schematic diagram of the *INV1* deletion strategy. **B.** DNA gel blot analysis of the *INV1* deletion mutants. **C.** Colony growth of wild-type strain P131, *INV1* deletion mutant *Δinv1-1* and *Δinv1-2*, and complemented strain *Δinv1/INV1* on OTA medium. **D.***M. oryzae* strains P131, *Δinv1-1, Δinv1-2,* and *Δinv1*/INV grown on MM or MM with different carbon sources (sucrose, glucose, and fructose), and the picture was taken at day 5 grown in 28°**C.** E. The mycelia fresh weight of strains P131, *Δinv1-1*, *Δinv1-2*, and *Δinv1*/INV grown in MM liquid media was measured without or with different carbon sources for 24 h. **F.** Colony growth of strains P131, *Δinv1-1, Δinv1-2*, and *Δinv1/INV1* on CM with glucose supplemented with the cell wall-disturbing agents 0.1 mg/ml CFW, 0.2 mg/ml CR, and 0.005% SDS. The cultures were incubated at 28℃ for 5 days before being photographed. **G.** Quantification of the growth reduction rates of mycelia growth on CM with glucose supplemented with cell wall-disturbing agents. The letters indicate significantly different groups (P < 0.001, one-way ANOVA with post-hoc Turkey tests) for the tested fungal strains. **H.** Rice spray assay. Rice leaves sprayed with conidium suspensions (1 × 10^5^ spores/ml) of the indicated strains were photographed at 5 dpi, and the relative lesion area was calculated. **I.** Quantification of relative lesion area in rice spraying assay as shown in panel H. **J.** Barley spray assay. Barley leaves sprayed with conidium suspensions (1 × 104 spores/ml) of the indicated strains were photographed at 5 dpi, and the relative lesion area was calculated. **K.** Quantification of relative lesion area in barley spraying assay as shown in panel **J.** Error bars denote standard deviations from three biological replicates with at least 9 plants. The letters indicate significantly different groups (P < 0.01, one-way ANOVA with post-hoc Turkey tests) for the tested fungal strains in infection assays. OTA, oatmeal–tomato agar; MM, minimal medium; CFW, Calcofluor white; CR, Congo red.

Supplementary Figure S4 **The characterization of *M. oryzae AMCase* gene function A.** Schematic diagram of the *AMCase* deletion strategy. **B.** DNA gel blot analysis of the *AMCase* deletion mutants. **C.** Colony growth of wild-type strain P131, *Δamcase-1*, and *Δamcase-2* on OTA medium. **D.** Quantification of colony diameter for wild-type P131, *Δamcase-1*, and *Δamcase-2* grown on OTA medium. **E.** Quantification of the sporulation of wild-type P131, *Δamcase-1*, and *Δamcase-2*. **F.***M. oryzae* strains P131, *Δamcase-1*, and *Δamcase-2* grown on MM with different carbon sources (glucose and chitin), and the picture was taken at day 5 grown in 28°C. **G.** Quantification of colony diameter for wild-type P131, *Δamcase-1*, and *Δamcase-2* grown on MM, MM with different carbon sources. Error bars denote standard deviations from three biological replicates. One-way ANOVA with post-hoc Turkey tests was applied but found no significance for the tested strains (*P* > 0.5). OTA, oatmeal–tomato agar; MM, minimal medium.

Supplementary Figure S5 **The phenotype of AMCase N-glycosylation single mutants A.** Colony growth of strains P131, *Δamcase-1*, *Δamcase/AMCase, N133G, N173G, N315G,* and *N381G* on CM medium supplemented with 0.1 mg/ml CFW. The cultures were incubated at 28℃ for 5 days before being photographed. **B.** Calculation of the growth reduction rates of mycelia growth on CM supplemented with CFW. **C.** Rice spraying assay. Rice leaves sprayed with conidium suspensions (1 × 10^5^ spores/ml) of the indicated strains were photographed at 5 dpi, and the relative lesion area was calculated. **D.** Quantification of relative lesion area in rice spraying assay as shown in panel C. **E.** Barley spraying assay. Barley leaves sprayed with conidium suspensions (1 × 10^4^ spores/ml) of the indicated strains were photographed at 5 dpi, and the relative lesion area was calculated. **F.** Quantification of relative lesion area in barley spraying assay as shown in panel E. **G.** Barley drop-inoculation assay. Barley leaves drop-inoculated with conidium suspensions (5 × 10^4^ spores/ml) of the indicated strains were photographed at 5 dpi, and the absolute lesion area was calculated. **H.** Quantification of lesion area in the barley drop-inoculation assay as shown in panel G. Error bars denote standard deviations from three biological replicates with at least 9 leaves. The letters indicate significantly different statistical groups (*P* < 0.01, one-way ANOVA with post-hoc Turkey tests) for those tested fungal strains.

Supplementary Figure S6 **The GFP intensity ratio of AMCase N-glycosylation mutants A.** GFP intensity ratio calculated for *Δamcase/AMCase*, *N133G*, *N173G*, *N315G*, *N381G*. **B.** GFP intensity ratio calculated for *N133G/N173G*, *N315G/N381G*, *N133G/N315G/N381G*, *N173G/N315G/N381G*, *N133G/N173G/N315G/N381G*. Error bars denote standard deviations from three biological replicates. The letters indicate significantly different statistical groups (*P* < 0.05, one-way ANOVA with post-hoc Turkey tests) for the tested fungal strains.
