# Supplementary figures and images for: Comparative Secretome Analysis of Magnaporthe oryzae Identified Proteins Involved in Virulence and Cell Wall Integrity
Source: Genomics Proteomics Bioinformatics. 2021 Jul 18;20(4):728–46. doi: 10.1016/j.gpb.2021.02.007 (PMC9880818; doi:10.1016/j.gpb.2021.02.007)

## Slide 1
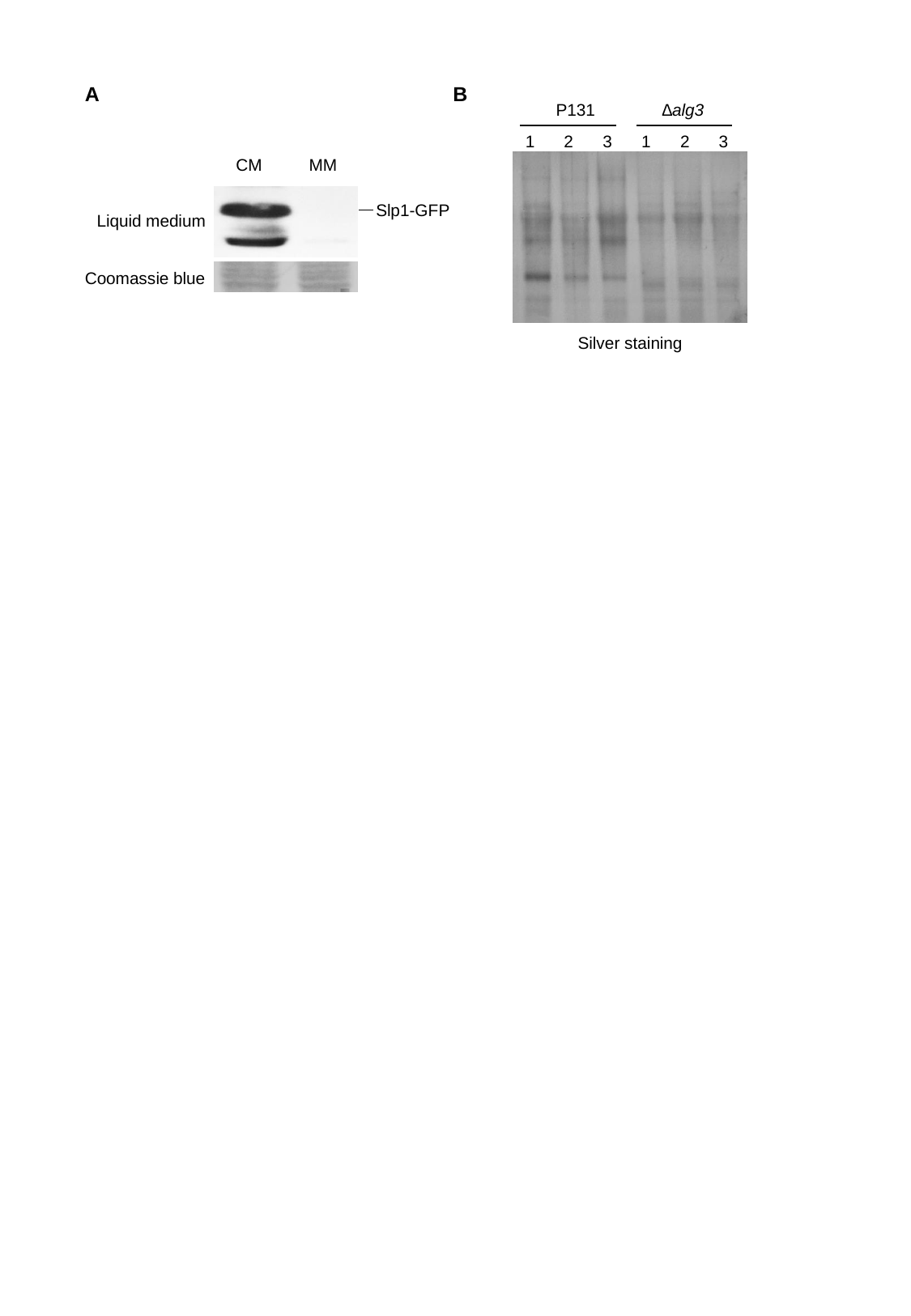

A
B
P131
∆alg3
1
2
3
1
2
3
Silver staining
CM
MM
 Slp1-GFP
Coomassie blue
Liquid medium

Supplement: Supplementary Figure S1 — Secreted proteins from M. oryzae P131 and Δalg3 strains A. Western blot shows the secretion of Slp1-GFP from P131 in CM and MM liquid medium; CM, nutrient-sufficient complete medium; MM, nutrient-deficient minimal medium. Coomassie blue stained gels were used as a loading control. B. Silver staining of secreted protein samples from the CM liquid culture of P131 and Δalg3 strains. Three replicates were performed for each strain. [file mmc1.pptx]
