# Supplementary material for: Comparative Secretome Analysis of Magnaporthe oryzae Identified Proteins Involved in Virulence and Cell Wall Integrity
Source: Genomics Proteomics Bioinformatics. 2021 Jul 18;20(4):728–46. doi: 10.1016/j.gpb.2021.02.007 (PMC9880818; doi:10.1016/j.gpb.2021.02.007)
Supplement: Supplementary Figure S2 — The tests for secreted proteins fused with a GFP tag A. Immunoblotting analysis of protein 03670-GFP, 10234-GFP, and 10318-GFP (group 3) from liquid medium and mycelial lysates from wild-type P131 and Δalg3 strains. B. GFP fluorescent signal was measured in 96-well plates (upper panel), and the ratio of GFP intensity from CM liquid culture against that from mycelial protein extracts was quantified (lower panel) for Slp1-GFP, MoGrp1-GFP, control GFP samples, and background control P131 samples. Error bars denote standard deviations from three biological replicates. The letters indicate significantly different groups (P < 0.001, one-way ANOVA with post-hoc Turkey tests) for the tested fungal strains. [file mmc2.pptx]

## Slide 1
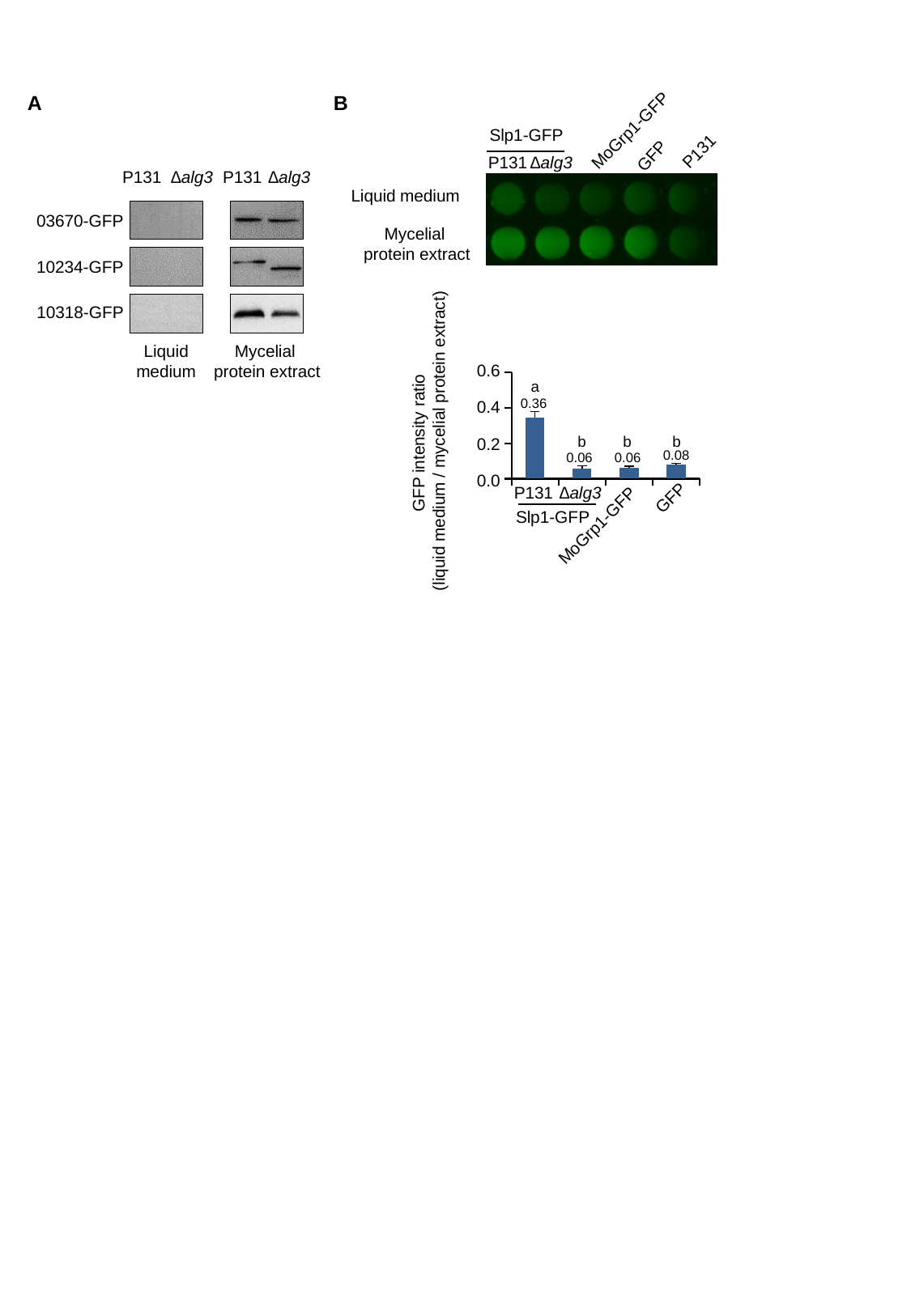

A
B
MoGrp1-GFP
Slp1-GFP
P131
GFP
P131
∆alg3
Liquid medium
Mycelial
protein extract
GFP intensity ratio
(liquid medium / mycelial protein extract)
0.6
### Chart
| Category | |
|---|---|
| slp131 | 0.3438403893878028 |
| slpko | 0.058998203484748046 |
| 7511gfp | 0.063402066452388 |
| gfp | 0.0775157004451922 |0.4
0.2
0.0
0.36
0.08
0.06
0.06
GFP
P131
∆alg3
Slp1-GFP
MoGrp1-GFP
a
b
b
b
P131
∆alg3
P131
∆alg3
03670-GFP
10234-GFP
10318-GFP
Liquid medium
Mycelial
protein extract
