# Supplementary material for: Comparative Secretome Analysis of Magnaporthe oryzae Identified Proteins Involved in Virulence and Cell Wall Integrity
Source: Genomics Proteomics Bioinformatics. 2021 Jul 18;20(4):728–46. doi: 10.1016/j.gpb.2021.02.007 (PMC9880818; doi:10.1016/j.gpb.2021.02.007)
Supplement: Supplementary Figure S3 — The characterization of M. oryzae INV1 function A. Schematic diagram of the INV1 deletion strategy. B. DNA gel blot analysis of the INV1 deletion mutants. C. Colony growth of wild-type strain P131, INV1 deletion mutant Δinv1-1 and Δinv1-2, and complemented strain Δinv1/INV1 on OTA medium. D.M. oryzae strains P131, Δinv1-1, Δinv1-2, and Δinv1/INV grown on MM or MM with different carbon sources (sucrose, glucose, and fructose), and the picture was taken at day 5 grown in 28°C. E. The mycelia fresh weight of strains P131, Δinv1-1, Δinv1-2, and Δinv1/INV grown in MM liquid media was measured without or with different carbon sources for 24 h. F. Colony growth of strains P131, Δinv1-1, Δinv1-2, and Δinv1/INV1 on CM with glucose supplemented with the cell wall-disturbing agents 0.1 mg/ml CFW, 0.2 mg/ml CR, and 0.005% SDS. The cultures were incubated at 28℃ for 5 days before being photographed. G. Quantification of the growth reduction rates of mycelia growth on CM with glucose supplemented with cell wall-disturbing agents. The letters indicate significantly different groups (P < 0.001, one-way ANOVA with post-hoc Turkey tests) for the tested fungal strains. H. Rice spray assay. Rice leaves sprayed with conidium suspensions (1 × 10^5^ spores/ml) of the indicated strains were photographed at 5 dpi, and the relative lesion area was calculated. I. Quantification of relative lesion area in rice spraying assay as shown in panel H. J. Barley spray assay. Barley leaves sprayed with conidium suspensions (1 × 10^4^ spores/ml) of the indicated strains were photographed at 5 dpi, and the relative lesion area was calculated. K. Quantification of relative lesion area in barley spraying assay as shown in panel J. Error bars denote standard deviations from three biological replicates with at least 9 plants. The letters indicate significantly different groups (P < 0.01, one-way ANOVA with post-hoc Turkey tests) for the tested fungal strains in infection assays. OTA, oatmeal–tomato agar; MM, mi [file mmc3.pptx]

## Slide 1
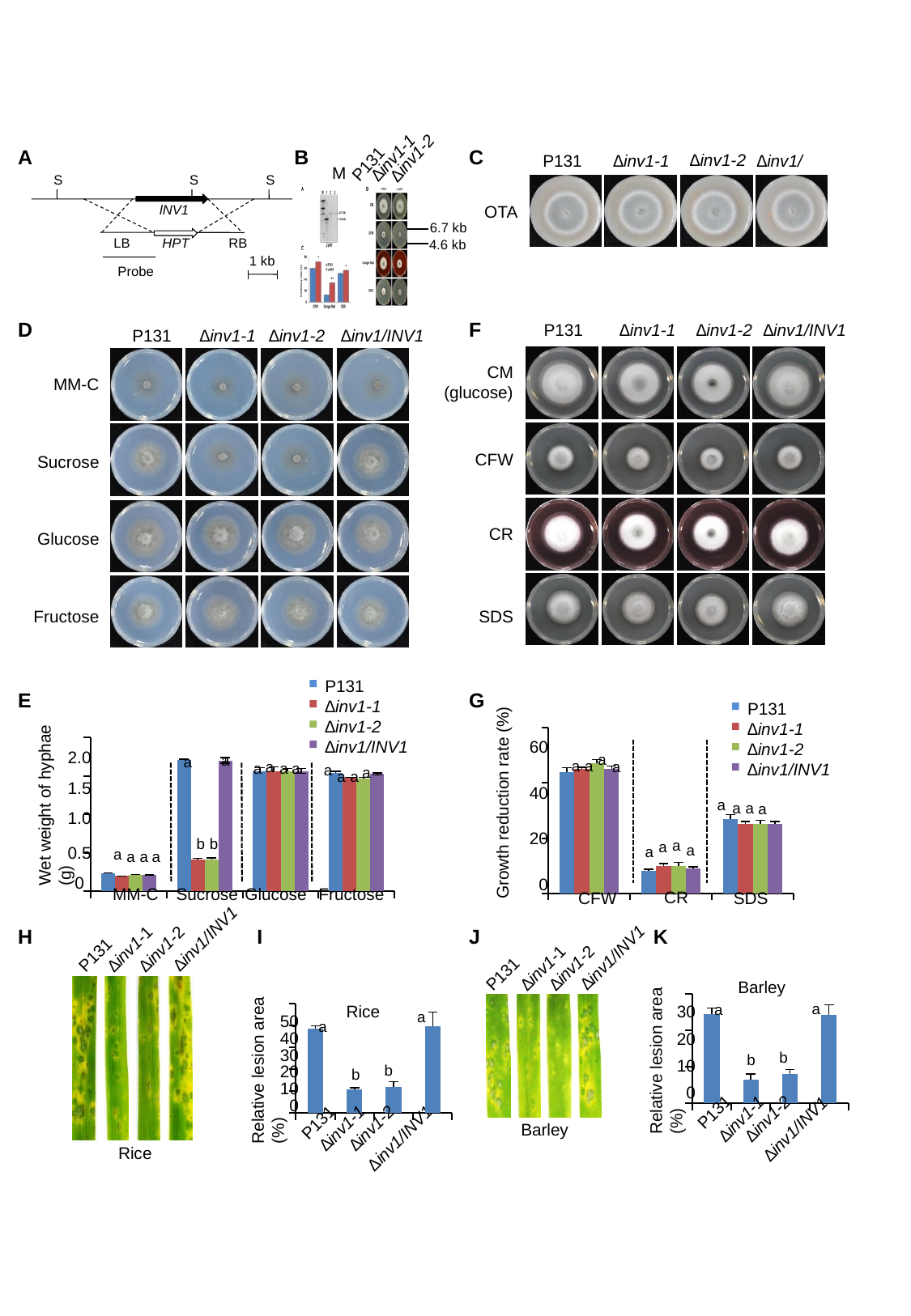

∆inv1-1
∆inv1-2
P131
M
6.7 kb
4.6 kb
A
B
C
∆inv1-2
P131
∆inv1/INV1
∆inv1-1
OTA
S
S
S
LB
RB
Probe
lNV1
HPT
1 kb
D
F
P131
∆inv1-1
∆inv1-2
∆inv1/INV1
CM
(glucose)
CFW
CR
SDS
P131
∆inv1-1
∆inv1-2
∆inv1/INV1
MM-C
Sucrose
Glucose
Fructose
P131
∆inv1-1
∆inv1-2
∆inv1/INV1
Wet weight of hyphae (g)
### Chart
| Category | P131 | MoInvKO1 | MoInvKO2 | cMoInv |
|---|---|---|---|---|
| no carbon | 0.23 | 0.2 | 0.213333333333333 | 0.213333333333333 |
| sucrose | 1.703333333333333 | 0.413333333333333 | 0.413333333333333 | 1.7 |
| glucose | 1.563333333333333 | 1.56 | 1.563333333333333 | 1.563333333333333 |
| fructose | 1.536666666666666 | 1.476666666666667 | 1.466666666666667 | 1.526666666666667 |2.0
1.5
1.0
0.5
0
 MM-C
Sucrose
Glucose
Fructose
a
a
a
a
a
a
a
a
a
a
b
b
a
a
a
a
E
G
Growth reduction rate (%)
P131
∆inv1-1
∆inv1-2
∆inv1/INV1
### Chart
| Category | P131 | 5785KO1 | 5785KO2 | 5785GFP |
|---|---|---|---|---|
| CFW | 0.44 | 0.45 | 0.47 | 0.45 |
| CR | 0.08 | 0.1 | 0.1 | 0.09 |
| SDS | 0.27 | 0.25 | 0.25 | 0.25 |60
40
20
0
CR
SDS
CFW
a
a
a
a
a
a
a
a
a
a
a
a
H
I
J
K
∆inv1/INV1
∆inv1-1
∆inv1-2
P131
Rice
∆inv1/INV1
∆inv1-1
∆inv1-2
P131
Barley
Relative lesion area (%)
Barley
### Chart
| Category | |
|---|---|
| P131 | 0.245070971909873 |
| KO1 | 0.0649656567872793 |
| KO2 | 0.0804947616696835 |
| GFP | 0.241604653855765 |30
20
10
0
P131
∆inv1-2
∆inv1-1
∆inv1/INV1
a
a
b
b
Relative lesion area (%)
Rice
### Chart
| Category | |
|---|---|
| P131 | 0.385561841954398 |
| KO1 | 0.106467768009609 |
| KO2 | 0.117024118341931 |
| GFP | 0.394192790805252 |50
40
30
20
10
0
P131
∆inv1-2
∆inv1-1
∆inv1/INV1
a
a
b
b
