# Supplementary material for: Comparative Secretome Analysis of Magnaporthe oryzae Identified Proteins Involved in Virulence and Cell Wall Integrity
Source: Genomics Proteomics Bioinformatics. 2021 Jul 18;20(4):728–46. doi: 10.1016/j.gpb.2021.02.007 (PMC9880818; doi:10.1016/j.gpb.2021.02.007)
Supplement: Supplementary Figure S4 — The characterization of M. oryzae AMCase gene function A. Schematic diagram of the AMCase deletion strategy. B. DNA gel blot analysis of the AMCase deletion mutants. C. Colony growth of wild-type strain P131, Δamcase-1, and Δamcase-2 on OTA medium. D. Quantification of colony diameter for wild-type P131, Δamcase-1, and Δamcase-2 grown on OTA medium. E. Quantification of the sporulation of wild-type P131, Δamcase-1, and Δamcase-2. F.M. oryzae strains P131, Δamcase-1, and Δamcase-2 grown on MM with different carbon sources (glucose and chitin), and the picture was taken at day 5 grown in 28°C. G. Quantification of colony diameter for wild-type P131, Δamcase-1, and Δamcase-2 grown on MM, MM with different carbon sources. Error bars denote standard deviations from three biological replicates. One-way ANOVA with post-hoc Turkey tests was applied but found no significance for the tested strains (P > 0.5). OTA, oatmeal–tomato agar; MM, minimal medium. [file mmc4.pptx]

## Slide 1
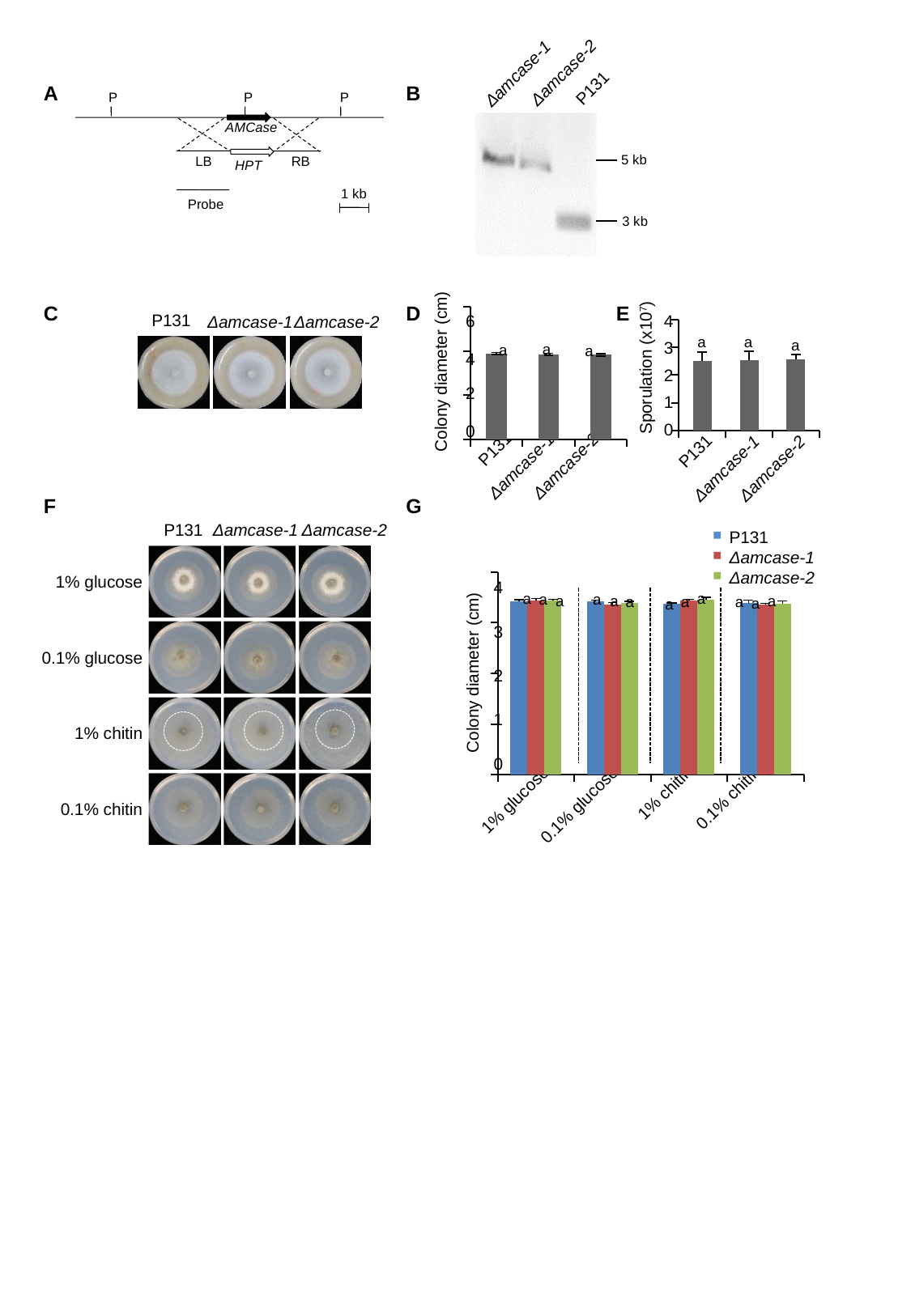

Δamcase-2
Δamcase-1
P131
5 kb
3 kb
A
B
P
P
P
AMCase
LB
RB
HPT
1 kb
Probe
### Chart
| Category | |
|---|---|
| P131 | 3.883333333333334 |
| KO1 | 3.85 |
| KO2 | 3.8249999999999997 |6
4
Colony diameter (cm)
2
0
P131
Δamcase-1
Δamcase-2
a
a
a
4
### Chart
| Category | |
|---|---|
| P131 | 2.515 |
| KO1 | 2.5500000000000003 |
| KO2 | 2.5749999999999997 |3
Sporulation (x107)
2
1
0
P131
Δamcase-1
Δamcase-2
a
a
a
C
D
E
P131
Δamcase-1
Δamcase-2
F
G
P131
Δamcase-1
Δamcase-2
1% glucose
0.1% glucose
1% chitin
0.1% chitin
P131
Δamcase-1
Δamcase-2
### Chart
| Category | P131 | 4732KO1 | 4732KO2 |
|---|---|---|---|
| 1% glucose | 3.4249999999999994 | 3.433333333333333 | 3.433333333333333 |
| 0.1% glucose | 3.416666666666666 | 3.358333333333334 | 3.383333333333333 |
| 1%chitin | 3.3666666666666667 | 3.433333333333333 | 3.4583333333333335 |
| 0.1%chitin | 3.3833333333333333 | 3.35 | 3.375 |4
3
Colony diameter (cm)
2
1
0
1% chitin
0.1% chitin
1% glucose
0.1% glucose
a
a
a
a
a
a
a
a
a
a
a
a
