# Supplementary material for: Comparative Secretome Analysis of Magnaporthe oryzae Identified Proteins Involved in Virulence and Cell Wall Integrity
Source: Genomics Proteomics Bioinformatics. 2021 Jul 18;20(4):728–46. doi: 10.1016/j.gpb.2021.02.007 (PMC9880818; doi:10.1016/j.gpb.2021.02.007)
Supplement: Supplementary Figure S5 — The phenotype of AMCase N-glycosylation single mutants A. Colony growth of strains P131, Δamcase-1, Δamcase/AMCase, N133G, N173G, N315G, and N381G on CM medium supplemented with 0.1 mg/ml CFW. The cultures were incubated at 28℃ for 5 days before being photographed. B. Calculation of the growth reduction rates of mycelia growth on CM supplemented with CFW. C. Rice spraying assay. Rice leaves sprayed with conidium suspensions (1 × 10^5^ spores/ml) of the indicated strains were photographed at 5 dpi, and the relative lesion area was calculated. D. Quantification of relative lesion area in rice spraying assay as shown in panel C. E. Barley spraying assay. Barley leaves sprayed with conidium suspensions (1 × 10^4^ spores/ml) of the indicated strains were photographed at 5 dpi, and the relative lesion area was calculated. F. Quantification of relative lesion area in barley spraying assay as shown in panel E. G. Barley drop-inoculation assay. Barley leaves drop-inoculated with conidium suspensions (5 × 10^4^ spores/ml) of the indicated strains were photographed at 5 dpi, and the absolute lesion area was calculated. H. Quantification of lesion area in the barley drop-inoculation assay as shown in panel G. Error bars denote standard deviations from three biological replicates with at least 9 leaves. The letters indicate significantly different statistical groups (P < 0.01, one-way ANOVA with post-hoc Turkey tests) for those tested fungal strains. [file mmc5.pptx]

## Slide 1
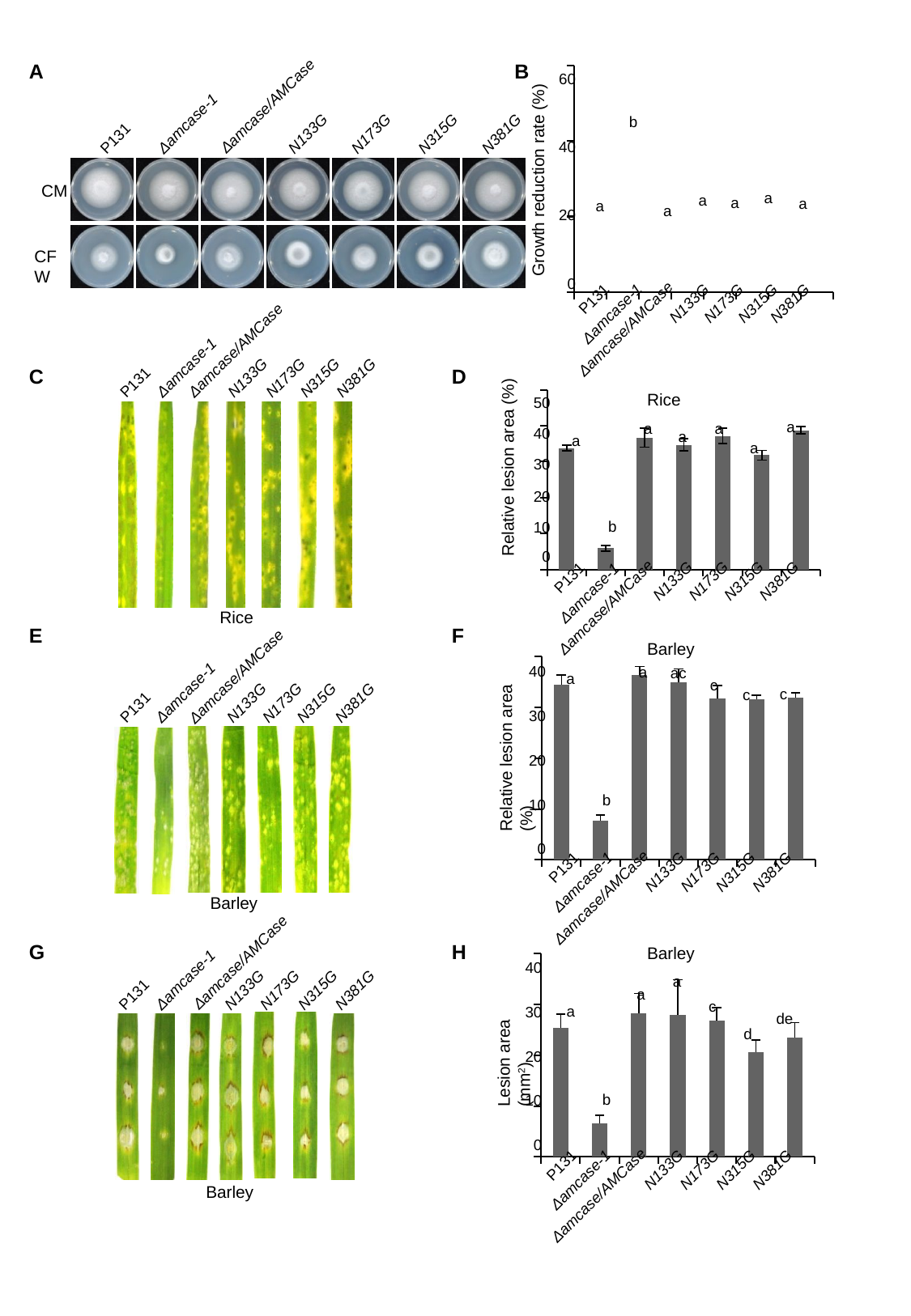

A
B
### Chart
| Category | |
|---|---|
| P131 | 0.17819706498951784 |
| 6B | 0.3983228511530399 |
| Z9 | 0.17555169848747842 |
| F | 0.20576131687242794 |
| H | 0.19449326303456352 |
| G | 0.21300935203740815 |
| I | 0.18704310562137638 |60
Growth reduction rate (%)
b
40
a
a
a
a
a
a
20
0
P131
N133G
N173G
N315G
N381G
Δamcase-1
Δamcase/AMCase
Δamcase/AMCase
Δamcase-1
N133G
N173G
N315G
N381G
P131
CM
CFW
Δamcase/AMCase
Δamcase-1
N133G
N173G
N315G
N381G
P131
Rice
C
D
Relative lesion area (%)
Rice
### Chart
| Category | |
|---|---|
| P131 | 0.3380672442244224 |
| 6B | 0.05879137233406125 |
| Z9 | 0.3668305204124635 |
| F | 0.34709292216652377 |
| H | 0.371858119159395 |
| G | 0.3178880548851108 |
| I | 0.3876098866285323 |50
a
a
a
40
a
a
a
30
20
b
10
0
P131
N133G
N173G
N315G
N381G
Δamcase-1
Δamcase/AMCase
E
F
Barley
### Chart
| Category | |
|---|---|
| 131 | 0.34519331661559943 |
| 6b | 0.07681479839572902 |
| z9 | 0.36426857849379024 |
| F | 0.34956388605363276 |
| H | 0.317242196345694 |
| G | 0.31632065923791025 |
| I | 0.31971869829012683 |40
Relative lesion area (%)
a
ac
a
c
c
c
30
20
b
10
0
P131
N133G
N173G
N315G
N381G
Δamcase-1
Δamcase/AMCase
Δamcase/AMCase
Δamcase-1
N133G
N173G
N315G
N381G
P131
Barley
G
H
Barley
### Chart
| Category | |
|---|---|
| P131 | 25.417466666666666 |
| 6b | 6.575622222222222 |
| z9 | 28.304388888888887 |
| F | 27.96242222222222 |
| H | 26.771111111111114 |
| G | 20.54441111111111 |
| I | 23.432044444444443 |40
a
Lesion area (mm2)
a
c
a
30
de
d
20
b
10
0
P131
N133G
N173G
N315G
N381G
Δamcase-1
Δamcase/AMCase
Δamcase/AMCase
Δamcase-1
N133G
N173G
N315G
N381G
P131
Barley
