# Supplementary material for: Comparative Secretome Analysis of Magnaporthe oryzae Identified Proteins Involved in Virulence and Cell Wall Integrity
Source: Genomics Proteomics Bioinformatics. 2021 Jul 18;20(4):728–46. doi: 10.1016/j.gpb.2021.02.007 (PMC9880818; doi:10.1016/j.gpb.2021.02.007)
Supplement: Supplementary Figure S6 — The GFP intensity ratio of AMCase N-glycosylation mutants A. GFP intensity ratio calculated for Δamcase/AMCase, N133G, N173G, N315G, N381G. B. GFP intensity ratio calculated for N133G/N173G, N315G/N381G, N133G/N315G/N381G, N173G/N315G/N381G, N133G/N173G/N315G/N381G. Error bars denote standard deviations from three biological replicates. The letters indicate significantly different statistical groups (P < 0.05, one-way ANOVA with post-hoc Turkey tests) for the tested fungal strains. [file mmc6.pptx]

## Slide 1
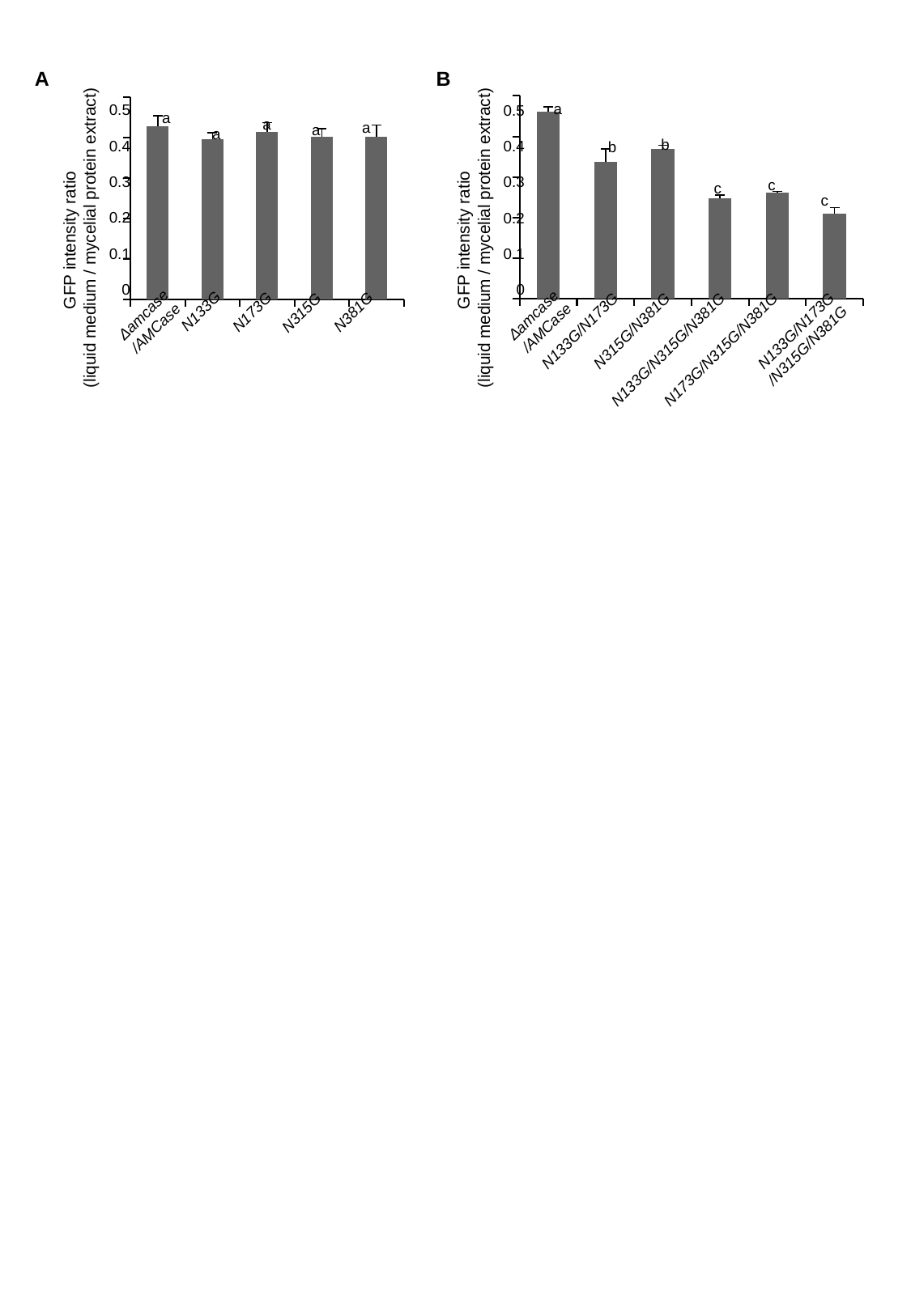

A
B
GFP intensity ratio
(liquid medium / mycelial protein extract)
### Chart
| Category | |
|---|---|
| z9 | 0.4277501385 |
| F | 0.395084309 |
| H | 0.413239946 |
| G | 0.40200199400000003 |
| I | 0.40206449450000004 |0.5
a
a
a
a
a
0.4
0.3
0.2
0.1
0
N133G
Δamcase
/AMCase
N173G
N381G
N315G
GFP intensity ratio
(liquid medium / mycelial protein extract)
### Chart
| Category | |
|---|---|
| z9 | 0.4605479973984275 |
| d | 0.33730041699060204 |
| e | 0.3695873070970892 |
| b | 0.2479306910797546 |
| c | 0.261168806782077 |
| a | 0.21004285152296254 |a
0.5
b
0.4
b
0.3
c
c
c
0.2
0.1
0
Δamcase
/AMCase
N133G/N173G
/N315G/N381G
N133G/N173G
N315G/N381G
N133G/N315G/N381G
N173G/N315G/N381G
