# Supplementary material for: Comparative Secretome Analysis of Magnaporthe oryzae Identified Proteins Involved in Virulence and Cell Wall Integrity
Source: Genomics Proteomics Bioinformatics. 2021 Jul 18;20(4):728–46. doi: 10.1016/j.gpb.2021.02.007 (PMC9880818; doi:10.1016/j.gpb.2021.02.007)
Supplement: Supplementary Table S3 [file mmc9.docx]

**Table S3 List of strains used in this study**

| Strain | Genotype description | Reference |
| --- | --- | --- |
| P131 | Wild type | [28] |
| *Δalg3* | *ALG3* deletion mutant in P131 | [28] |
| MoGrp1GFP | *MoGrp1-GFP* transformant of P131 | [30] |
| *ΔInv1* | *Inv1* deletion mutant of P131 | This study |
| *ΔInv1/INV1* | *Inv1* complement of *ΔInv1* | This study |
| *ΔAMCase* | *AMCase* deletion mutant of P131 | This study |
| *ΔAMCase/AMCase* | *AMCase* complement of *ΔAMCase* | This study |
| P131/Slp1-GFP | *Slp1-GFP* transformant of P131 | This study |
| *Δalg3*/Slp1-GFP | *Slp1-GFP* transformant of *Δalg3* | This study |
| P131/05785-GFP | *05785-GFP* transformant of P131 | This study |
| *Δalg3*/05785-GFP | *05785-GFP* transformant of *Δalg3* | This study |
| P131/01956-GFP | *01956-GFP* transformant of P131 | This study |
| *Δalg3*/01956-GFP | *01956-GFP* transformant of *Δalg3* | This study |
| P131/08772-GFP | *08772-GFP* transformant of P131 | This study |
| *Δalg3*/08772-GFP | *08772-GFP* transformant of *Δalg3* | This study |
| P131/03826-GFP | *03826-GFP* transformant of P131 | This study |
| *Δalg3*/03826-GFP | *03826-GFP* transformant of *Δalg3* | This study |
| P131/09460-GFP | *09460-GFP* transformant of P131 | This study |
| *Δalg3*/09460-GFP | *09460-GFP* transformant of *Δalg3* | This study |
| P131/10209-GFP | *10209-GFP* transformant of P131 | This study |
| *Δalg3*/10209-GFP | *10209-GFP* transformant of *Δalg3* | This study |
| P131/10466-GFP | *10466-GFP* transformant of P131 | This study |
| *Δalg3*/10466-GFP | *10466-GFP* transformant of *Δalg3* | This study |
| P131/13764-GFP | *13764-GFP* transformant of P131 | This study |
| *Δalg3*/13764-GFP | *13764-GFP* transformant of *Δalg3* | This study |
| P131/00592-GFP | *00592GFP* transformant of P131 | This study |
| *Δalg3*/00592-GFP | *00592-GFP* transformant of *Δalg3* | This study |
| P131/04732-GFP | *04732-GFP* transformant of P131 | This study |
| *Δalg3*/04732-GFP | *04732-GFP* transformant of *Δalg3* | This study |
| P131/03670-GFP | *03670-GFP* transformant of P131 | This study |
| *Δalg3*/03670-GFP | *03670-GFP* transformant of *Δalg3* | This study |
| P131/10234-GFP | *10234-GFP* transformant of P131 | This study |
| *Δalg3*/10234-GFP | *10234-GFP* transformant of *Δalg3* | This study |
| P131/10318-GFP | *10318-GFP* transformant of P131 | This study |
| *Δalg3*/10318-GFP | *10318-GFP* transformant of *Δalg3* | This study |
