# Supplementary material for: Comparative Secretome Analysis of Magnaporthe oryzae Identified Proteins Involved in Virulence and Cell Wall Integrity
Source: Genomics Proteomics Bioinformatics. 2021 Jul 18;20(4):728–46. doi: 10.1016/j.gpb.2021.02.007 (PMC9880818; doi:10.1016/j.gpb.2021.02.007)
Supplement: Supplementary Table S4 [file mmc10.docx]

**Table S4 List of oligonucleotide primers used in this study**

| Name | Sequence (5'- 3') | Application |
| --- | --- | --- |
| 03641promoter F | CGGAATTCTGGCTCTGGATACTGAAGGC | 03641 promoter |
| 03641promoter R | CGGGATCCTTTGGCGGTTTGGTGCTC | 03641 promoter |
| 05785F | caccaaaccgccaaaggatccATGAAATTCACATTTGTGTCATCGG | 05785-GFP |
| 05785R | acctctagaactagtggatccCCATCCGTTCCACCAGGTG | 05785-GFP |
| 01956F | caccaaaccgccaaaggatccATGAAGGCTACCTTTGTTACCCTC | 01956-GFP |
| 01956R | acctctagaactagtggatccCTGGAGTGTGACCTTTCCGC | 01956-GFP |
| 08772F | caccaaaccgccaaaggatccATGAAGGCAACAATCCTTACACTG | 08772-GFP |
| 08772R | acctctagaactagtggatccCAACAACATAGTAAGC | 08772-GFP |
| 03826F | caccaaaccgccaaaggatccATGCATCATCGCCGCCAT | 03826-GFP |
| 03826R | acctctagaactagtggatccAACCACTCTCAACACGGCATTAC | 03826-GFP |
| 07949F | caccaaaccgccaaaggatccATGGGCTACTACACCGACCACC | 07949-GFP |
| 07949R | acctctagaactagtggatccATCGCTGTCAGAGTCATTGTCG | 07949-GFP |
| 09460F | caccaaaccgccaaaggatccATGCAGCTCTCAAGCCTCCTC | 09460-GFP |
| 09460R | acctctagaactagtggatccAGTGGGTATGTAATGACAATGTCTGTT | 09460-GFP |
| 10209F | caccaaaccgccaaaggatccATGTTCTTCTTCAAAGTCTTGGTCG | 10209-GFP |
| 10209R | acctctagaactagtggatccCTTGACAAACTTGTACTGAACCGTC | 10209-GFP |
| 10466F | caccaaaccgccaaaggatccATGAAGGCTACGACGCTTTACAT | 10466-GFP |
| 10466R | acctctagaactagtggatccTCCGCTGCAACTGAATTCG | 10466-GFP |
| 13764F | caccaaaccgccaaaggatccATGCGTTCCTCGGTTGGTT | 13764-GFP |
| 13764R | acctctagaactagtggatccCTGACGCGGCATGTAGCG | 13764-GFP |
| 00592F | caccaaaccgccaaaggatccATGTACACAAAGACCAT | 00592-GFP |
| 00592R | acctctagaactagtggatccCAGCATGAAGTAACCCA | 00592-GFP |
| 04732F | caccaaaccgccaaaggatccATGTCACTCGTTAACCTCTCCAGG | 04732-GFP |
| 04732R | acctctagaactagtggatccCAGACCAACGGATCCGCG | 04732-GFP |
| 05785LBF | GGACTAGTGGTTTGAGTCATTGGCATC | *INV1* knockout |
| 05785LBR | CGGAATTCGGGAGCGGAACAGATTACA | *INV1* knockout |
| 05785RBF | CCATCGATCAGTCTCACTTGTGTCGTTGTC | *INV1* knockout |
| 05785RBR | AAGGGCCCGCCAGAAGAAGTAAGGTGAAGT | *INV1* knockout |
| 04732LBF | CGGGATCCCCCTTTCCAAGCCATTCCGT | *AMCase* knockout |
| 04732LBR | CGGAATTCCGATTTGCACACTTGGGATGG | *AMCase* knockout |
| 04732RBF | CCCAAGCTTTGGCGCAAGTTCGTCTGTAA | *AMCase* knockout |
| 04732RBR | CCATCGATGCACCTCCTCGTTGTACCAG | *AMCase* knockout |
| 04732UP | TAACCGCCCCTTTGTTAGCC | *AMCase* L check |
| HPTUP | GACAGACGTCGCGGTGAGTT | *AMCase* L check |
| 04732DOWN | GTGCGTCCAAACACCTTGTA | *AMCase* R check |
| HPTDOWN | TCTGGACCGATGGCTGTGTAG | *AMCase* R check |
| 04732 check F | ATGTCACTCGTTAACCTC | *AMCase* gene check |
| 04732 chevk R | CAGACCAACGGATCCGCG | *AMCase* gene check |
| 04732 mutant F | aggactccagcaaacggatccATGTCACTCGTTAACCTCTCCAGG | *AMCase* point mutation |
| 04732 mutant R | acctctagaactagtggatccCAGACCAACGGATCCGCG | *AMCase* point mutation |
| 04732 (133)F | AGC GCCGAGGGCC GCACGGCGTT CGTCAAGAC | *AMCase* point mutation |
| 04732(133)R | GCCGTGCGGC CCTCGGCGCT GCCCACGTTG CT | *AMCase* point mutation |
| 04732(173)F | CAACGCCATC AGCCCCGGCG | *AMCase* point mutation |
| 04732(173)R | GATGGCGTTG CACCCGAGGC | *AMCase* point mutation |
| 04732(315)F | CCCAAGA ACGGCGGCAC CCGCCGGCAGGGCAG | *AMCase* point mutation |
| 04732(315)R | CGGGTGCCGC CGTTCTTGGG GTAGGCGCTC AG | *AMCase* point mutation |
| 04732(381)F | ACAACGGCGC CACCAGCTGG | *AMCase* point mutation |
| 04732(381)R | GCGCCGTTGT AGACAAAGGG | *AMCase* point mutation |
